# Cutting Force Modeling and Experimental Investigation of Axial Ultrasonic Vibration-Assisted Milling for Titanium Alloy Ti-6Al-4V

**DOI:** 10.3390/ma19194084

**Published:** 2026-09-24

**Authors:** Xubo Li, Cunqiang Zang, Shihao Zhang, Kang Jin, Jiaqi Jiang, Zhenting Li

**Affiliations:** 1 College of Mechanical Engineering, Baoji University of Arts and Sciences, Baoji 721016, China; 17560381680@163.com (C.Z.);; 2 Shaanxi Province Key Laboratory of Advanced Manufacturing and Evaluation of Robot Key Components, Baoji University of Arts and Sciences, Baoji 721016, China

**Keywords:** ultrasonic vibration-assisted milling, milling force model, contact rate model, finite element simulation

## Abstract

Titanium alloy (Ti-6Al-4V) exhibits exceptional comprehensive properties, which have resulted in its widespread adoption across diverse industrial sectors. However, its poor machinability poses a significant bottleneck to achieving high-efficiency and high-precision manufacturing. To address the challenges inherent in conventional milling (CM), axial ultrasonic vibration is introduced at the end of the milling spindle to enhance machining quality. Through kinematic analysis, a model of the tool-tip trajectory and tool-workpiece contact rate is established. By incorporating the vibration displacement vector, an instantaneous cutting thickness equation is derived. Subsequently, by integrating tool geometric parameters, instantaneous cutting thickness (IUCT), contact rate, and vibration impact effects, an analytical prediction model for transient cutting forces in ultrasonic vibration-assisted milling (UVAM) is developed. Concurrently, a finite element (FE) model is constructed to simulate the milling process, enabling numerical analysis of chip morphology, stress–strain fields, and the evolution of cutting forces. Finally, a test platform is established to verify the reliability and predictive accuracy of the proposed model. Comparative analysis reveals the advantages of UVAM over CM and elucidates the roles of UVAM parameters in machining performance. The results indicate that the experimentally measured tri-axial milling forces align well with the analytical model predictions and FE simulation outputs, exhibiting consistent trends. Compared with CM, axial UVAM reduces the tri-axial average cutting forces by 14.73%, 15.47%, and 14.49%, respectively, while simultaneously decreasing the surface roughness Ra by approximately 12.1%, indicating an improvement in surface quality. This research provides a reference for understanding the influence of process parameters and a basis for subsequent multi-parameter coupled optimization in the high-performance machining of difficult-to-machine materials.

## 1. Introduction

Titanium alloy (Ti-6Al-4V), widely recognized as a quintessential hard-to-process material, is extensively utilized in the aerospace, biomedical, and automotive sectors attributed to its low density, high strength-to-density ratio, and good thermal stability [1]. Nevertheless, its typical attributes, which include a low elastic modulus, strong chemical affinity leading to significant spring-back, and a propensity for chatter, render it notoriously challenging to machine [2,3,4]. During conventional milling (CM) of Ti-6Al-4V, overly elevated cutting temperatures can accelerate tool diffusion wear and alter the workpiece surface integrity, resulting in diminished machining accuracy and surface quality. Furthermore, severe work hardening significantly complicates subsequent processing steps, exacerbates tool flank wear, and shortens tool life, hence complicating the fulfillment of the rigorous standards for high-end components. These issues collectively contribute to low machining efficiency and elevated costs, severely limiting the widespread industrial application of titanium alloys. With the advent of advanced manufacturing technologies, ultrasonic vibration-assisted milling (UVAM) has proven to be a compelling solution. This technique reduces cutting stress, workpiece deformation, and cutting heat generation, while also possessing vibration-damping and chatter-suppression capabilities, ultimately improving machining precision [5]. By superposition of high-frequency ultrasonic vibrations onto the cutting tool or workpiece, the tool-workpiece engagement state is fundamentally altered, enabling high-frequency intermittent cutting. Given its recognized effectiveness in elevating surface quality and reducing cutting forces, UVAM has garnered significant research interest and has been applied to titanium alloys [6]. This unique cutting mode not only enables the feasible and convenient processing of materials, but also improves the dimensional precision and surface integrity of parts, with additional merits such as low surface roughness, reduced cutting forces, and favorable chip breakability. Therefore, in-depth research on UVAM of titanium alloys is of substantial practical worth for encouraging the wide adoption of titanium alloys across various industrial fields, enhancing machining quality and efficiency, and reducing production costs [7,8].

Nevertheless, the mechanical behavior mechanism of UVAM is relatively complex, and relevant studies on milling force modeling remain insufficient. Current modeling methods for cutting forces mainly include empirical models [9], analytical models [10,11], finite element simulation models [12] and mechanistic models [13,14]. Accordingly, various theoretical models and numerical simulation methodologies have been proposed in previous studies.

Regarding research on UVAM force models, Endres et al. [15] early proposed a dual-mechanism (shear and plowing) method for cutting force prediction, established corresponding mechanical models and coefficient calibration procedures, and verified model accuracy through cutting tests. Subsequently, Altintas [16] established a mathematical formulation of oblique cutting forces, providing a theoretical basis for determining parameters such as βn and ϕn. Lin et al. [17] decomposed helical cutting edges into micro-element oblique cutting units based on axial discretization, revealed the influence of tool geometry and kinematic parameters on micro-element forces, and verified the model accuracy through experiments. Grossi et al. [18] adopted dynamic compensation technology to realize the cutting force coefficient calibration over a wide speed range; however, their method relied on customized compensation filtering and failed to fully consider the nonlinear material effects. Campatelli et al. [19] explored the effects of feed per tooth and cutting velocity on milling force coefficients of aluminum alloys based on Altintas’ dual-mechanism mechanistic model via response surface methodology, and improved prediction accuracy by introducing tool runout correction. Nevertheless, their research mainly focused on tangential force coefficients, with relatively insufficient analysis of axial and radial force parameters. Additionally, Wang et al. [20] presented a force coefficient estimation approach for ball-end mills that takes both average value and amplitude into account. Moufki et al. [21] established an analytical model for peripheral milling forces of Ti-6Al-4V based on the mechanical analytical theory of oblique cutting, which can reduce calibration experiments; nevertheless, the steady-state chip assumption leads to significant errors under large feed and small rake angle conditions. Wan et al. [22] proposed a novel micro-milling force prediction method considering the effects of uncut chip thickness, but it is still based on the ideal steady-state cutting assumption and provides an inadequate description of the dynamic machining process. Li et al. [23] investigated the tool-workpiece contact rate and milling force modeling in elliptical UVAM, established an analytical contact rate model and an instantaneous milling force prediction model, and verified the favorable impact attained through elliptical vibration on contact state and milling force through experiments. Duan et al. [24] established a semi-empirical force model under nano-biological lubrication conditions and verified its accuracy in cutting force prediction. However, their work was based on mathematical statistics and lacked theoretical analysis of the chip-removal mechanism.

Most existing milling force models are based on steady-state or quasi-steady-state assumptions under CM conditions, usually adopting fixed cutting-force coefficients or simplified linear chip-thickness relationships. Such approaches cannot fully describe the periodic tool-workpiece contact-separation behavior, dynamic chip-thickness fluctuation, and instantaneous cutting parameter changes induced by axial ultrasonic vibration. Especially under machining conditions involving multi-parameter coupling, the prediction accuracy and engineering applicability of these models show obvious limitations. To address the above issues, this study adopts a technical approach combining theoretical modeling, finite element analysis, and experimental verification to systematically establish and validate a cutting force prediction model for UVAM. First, based on vibration milling theory, the tool motion trajectory during machining is plotted. Combined with the kinematics of ultrasonic vibration milling and tool geometric parameters, an analytical milling force model considering instantaneous dynamic chip thickness and tool-workpiece contact rate is established. Meanwhile, a three-dimensional finite element model of axial UVAM is constructed to simulate and analyze milling forces, stress–strain behavior in the cutting zone, and the chip formation process. Finally, an axial UVAM experimental platform is set up. The prediction capability of the model is evaluated by comparing the analytical model results, FE simulation results, and measured milling forces. On this basis, the influences of spindle speed, feed rate, ultrasonic amplitude, and vibration frequency on milling force and chip morphology are further investigated. The findings of this study contribute to the theoretical understanding of parameter effects and provide a practical reference for subsequent multi-parameter optimization in machining difficult-to-machine materials.

## 2. Kinematic and Contact State Analysis

UVAM is a high-efficiency hybrid machining technology derived from conventional milling processes. Its working principle is illustrated in Figure 1. The key concept is to introduce high-frequency ultrasonic vibration (above 10 kHz) to the tool or workpiece end via a dedicated vibration system, so that the cutting process is superimposed with high-frequency periodic reciprocating vibration in addition to the original milling main motion and feed motion [25].

In conventional milling, the trajectory of a given cutting edge is the vector resultant of the tool’s rotational motion and its linear feed motion, resulting in a trochoidal path. Thus, in the absence of ultrasonic vibration, the motion equations for the tool center are given by:

(1) $$\left\{\begin{array}{c} x(t)=x_{0}+V_{f}\cdot t\\ y(t)=y_{0}\\ z(t)=z_{0} \end{array}\right.$$

where *x*_0_, *y*_0_ and *z*_0_ are the initial coordinates of the tool center; *V*_f_ and *t* represent the feed speed and cutting time, respectively.

The trajectory equation of the *j*-th tool tip is:

(2) $$\left\{\begin{array}{c} x_{j}=x_{0}+V_{f}\cdot t+R\sin(\frac{2\pi n}{60}t+\frac{2\pi j}{N})\\ y_{j}=y_{0}+R\cos(\frac{2\pi n}{60}t+\frac{2\pi j}{N})\\ z_{j}=z_{0} \end{array}\right.$$

where *R* denotes the tool radius, *n* represents the spindle speed, *N* represents how many cutter teeth there are, and *j* denotes the *j*-th cutter tooth.

The defining distinction between UVAM and CM is the superimposed ultrasonic vibration on the cutting tool. This study employs axial UVAM, in which a high-frequency vibration is applied along the tool axis. Therefore, the tool center motion in axial UVAM is described as follows:

(3) $$\left\{\begin{array}{c} x(t)=x_{0}+V_{f}\cdot t\\ y(t)=y_{0}\\ z(t)=z_{0}+A\sin(2\pi ft+\varphi ) \end{array}\right.$$

Consequently, the trajectory of the *j*-th cutting edge is given by:

(4) $$\left\{\begin{array}{c} x_{j}=x_{0}+V_{f}\cdot t+R\sin(\frac{2\pi n}{60}t+\frac{2\pi j}{N})\\ y_{j}=y_{0}+R\cos(\frac{2\pi n}{60}t+\frac{2\pi j}{N})\\ z_{j}=z_{0}+A\sin(2\pi ft+\varphi ) \end{array}\right.$$

where *f* is the oscillation frequency, *A* is the vibration amplitude, and φ is the initial phase angle.

To intuitively reveal the influence of axial ultrasonic vibration on the spatial motion characteristics of the tool tip, based on the above kinematic equations, MATLAB R2023a software was used to solve the three-dimensional tool tip trajectories under CM and axial UVAM conditions, respectively, and a comparative analysis was performed under the same process parameters. The results are shown in Figure 2.

In axial UVAM, the superposition of high-frequency axial oscillations onto the rotational and feed motions imparts a periodic variation in the instantaneous relative velocity and contact conditions at the tool-workpiece boundary, engendering an intermittent cutting process characterized by alternating contact and separation phases. Since material removal primarily occurs at the side edge of the tool, a local coordinate system was established for an infinitesimal element of a single cutting edge, as shown in Figure 3. The resultant velocity is decomposed along the helical cutting edge and its normal direction. The X-axis is oriented along the cutting edge, while the Y-axis is perpendicular to it. This local framework serves as the kinematic basis for the subsequent derivation of the tool-workpiece separation criterion and the contact rate model.

For up-milling, the velocity component along the Y-direction can be expressed as:

(5) $$V_{y}=(V_{c}+V_{f})\cos\beta +V_{z}\sin\beta$$

Here, *V*_z_ represents the axial vibration velocity, as defined by Equation (3):

(6) $$V_{z}=2\pi Afcos\left(2\pi ft\right)$$

The cutting speed is denoted by *V*_c_:

(7) $$V_{c}=\frac{\pi dn}{1000}$$

and *V*_f_ is the feed speed:

(8) $$V_{f}=nf_{z}N$$

where *f*_z_ is the feed per tooth, which satisfies the relationship with the feed per revolution *f*_r_: *f*_r_ = *f*_z_*N*, where *N* is the number of teeth on the milling cutter. This study used a four-edge end mill with *N* = 4.

It is known that the introduction of axial ultrasonic vibration causes the milling cutter to reciprocate up and down; hence, the value and orientation of the axial vibration velocity *V*_z_ change continuously, which in turn leads to dynamic variation in *V*_y_. Under this circumstance, the state of interaction between the cutting tool and the workpiece can be partitioned into the following three types:

(1) *V*_y_ < 0: Tool-workpiece separation occurs, leading to intermittent cutting;
(2) *V*_y_ = 0: Critical separation state;
(3) *V*_y_ > 0: No separation, continuous contact.

The Tool-Workpiece Contact Rate *R*(*t*) is defined as the proportion of time within a single vibration period during which the tool and workpiece are in actual contact [26].

The variation in *V*_y_ and the associated separation state are depicted in Figure 4.

Considering *t*_1_ to *t*_4_ as one vibration period, *t*_1_ is the separation point, and *t*_2_ is the re-contact point. The interval (*t*_1_, *t*_2_) represents the separation phase, while (*t*_2_, *t*_4_) represents the cutting phase. The times *t*_1_ and *t*_2_ satisfy:

(9) $$V_{y}=(V_{c}+V_{f})\cos\beta +V_{z}\sin\beta =0$$

The *R*(*t*) for this period is defined as:

(10) $$R(t)=\frac{T-(t_{2}-t_{1})}{T}=1-(t_{2}-t_{1})f$$

By combining the above equations, the *R*(*t*) model can be obtained:

(11) $$R(t)=1-\frac{1}{\pi }\left[\cos^{-1}\left(\frac{\left(v_{c}+v_{f}\right)\cot\beta }{2\pi fA}\right)\right]$$

Equation (11) reveals that *R*(*t*) is not constant but is governed by several process variables, namely feed rate, spindle speed, vibration frequency, and ultrasonic amplitude. These interrelated parameters collectively govern the tool-workpiece contact behavior.

## 3. Cutting Force Modeling

Figure 5 provides a schematic representation of the tool motion and cutting forces during axial UVAM. For an arbitrary axial micro-element of the cutting edge, the local cutting force can be resolved into tangential (*dF*_T_), radial (*dF*_R_), and axial (*dF*_A_) components.

In CM, the instantaneous uncut chip thickness (IUCT) is determined by the feed per tooth, tooth angular position, and tool geometric parameters, showing periodic variation. In axial UVAM, however, high-frequency axial vibration dynamically changes the trajectories of adjacent teeth and their contact state, leading to high-frequency fluctuation of chip thickness, which directly affects the material removal volume and cutting load. Therefore, its accurate modeling is of vital importance. Existing models often treat the tool as a rigid body and fail to fully consider the effect of tool-tip trajectory deviations caused by ultrasonic vibration on chip thickness. Based on the principle of relative kinematics, this study incorporates the vibration displacement vector into the calculation of the tool tip trajectory to construct a dynamic instantaneous chip-thickness model. Figure 6 shows the formation schematic of IUCT in axial UVAM. As shown in the locally enlarged area, the shaded area between the present tooth trajectory and the preceding adjacent tooth trajectory represents the uncut chip region, and its normal thickness is defined as the effective IUCT.

The instantaneous angular position of the j-th tooth at an axial depth *z* is:

(12) $$\theta_{j}(t)=\omega t-\frac{2\pi (j-1)}{N}-\frac{z\tan\beta }{R}$$

with *β* as the helix angle, and (ztanβ)/R is the axial lag angle caused by the helical cutting edge.

The (j − 1)-th adjacent tooth passes through the same angular position earlier by a time interval Δ*t*, and its corresponding axial vibration displacement at time *t* is:

(13) $$z_{s,j-1}(t)=A\sin\left(\omega (t-\Delta t)+\varphi_{0}\right)$$

The axial displacement difference between adjacent teeth at the same time and axial position is:

(14) $$\Delta z(t)=z_{s,j}(t)-z_{s,j-1}(t)=A\left[\sin(\omega t+\varphi_{0})-\sin(\omega (t-\Delta t)+\varphi_{0})\right]$$

Using trigonometric identities, this simplifies to:

(15) $$\Delta z(t)=2A\sin\left(\frac{\omega \Delta t}{2}\right)\cos\left(\omega (t-\frac{\Delta t}{2})+\varphi_{0}\right)$$

The frequency ratio k=60f/nN, which relates the ultrasonic vibration frequency to the tooth-passing frequency, was thus defined, yielding:

(16) $$\Delta z(t)=2A\sin(\pi k)\cos\left(\omega t-k\pi +\varphi_{0}\right)$$

The parameter k reflects the number of ultrasonic vibration cycles experienced within the passing time of adjacent cutter teeth, and determines the vibration phase difference between adjacent cutter teeth.

Without considering tool runout and regenerative vibration, the geometric uncut chip thickness in CM can be expressed as:

(17) $$t_{\mathrm{CD}}=f_{z}\sin\theta_{j}(t)$$

In view of the helical design of the cutter’s cutting edge, the axial displacement difference is converted into the chip-thickness variation normal to the cutting edge. Finally, the instantaneous chip thickness *t*_UD_ of axial UVAM is obtained as:

(18) $$t_{\mathrm{UD}}=f_{z}\sin\theta_{j}(t)+\Delta z(t)\sin\beta$$

Since material removal is conditional upon geometric engagement, tool-workpiece contact, and positive chip thickness, the effective IUCT is:

(19) $$t_{\mathrm{UD}}^{eff}=\max(0,t_{\mathrm{UD}})$$

The milling cutter is discretized into several micro-element cutting units. Assuming that each tool micro-element has the same stress conditions, the length of the tool micro-element is denoted as dz. Based on the oblique-cutting principle and cutting mechanics theory, a numerical analysis model is established. Each micro-element is treated as an individual oblique cutting process. The forces generated in each direction are calculated by a numerical method and then integrated to obtain the total milling forces of the cutter during the milling process.

(20) $$\left\{\begin{array}{l} dF_{T}=(K_{\mathrm{TC}}t_{\mathrm{UD}}^{eff}+K_{\mathrm{TE}})\cdot dz\\ dF_{R}=(K_{\mathrm{RC}}t_{\mathrm{UD}}^{eff}+K_{\mathrm{RE}})\cdot dz\\ dF_{A}=(K_{\mathrm{AC}}t_{\mathrm{UD}}^{eff}+K_{\mathrm{AE}})\cdot dz \end{array}\right.$$

in which *K*_TC_, *K*_RC_, and *K*_AC_ are the tangential, radial, and axial cutting force coefficients, respectively; *K*_TE_, *K*_RE_, *K*_AE_ are the corresponding edge force coefficients; and the elemental cutting forces in the tangential, radial, and axial directions are denoted by *dF*_T_, *dF*_R_, *dF*_A_.

Figure 7 depicts the oblique cutting model. The oblique cutting theory yields the modified cutting force coefficients [27], which are:

(21) $$\left\{\begin{array}{l} K_{\mathrm{TC}}=\frac{\tau_{s}}{\sin\phi_{n}}\frac{\cos\left(\beta_{n}-\gamma_{n}\right)+\tan\lambda_{s}\tan\eta_{c}\sin\beta_{n}}{\sqrt{\cos^{2}\left(\phi_{n}+\beta_{n}-\gamma_{n}\right)+\tan^{2}\eta_{c}\sin^{2}\beta_{n}}}\\ K_{\mathrm{RC}}=\frac{\tau_{s}}{\sin\phi_{n}}\frac{\cos\left(\beta_{n}-\gamma_{n}\right)\tan\lambda_{s}-\tan\eta_{c}\sin\beta_{n}}{\sqrt{\cos^{2}\left(\phi_{n}+\beta_{n}-\gamma_{n}\right)+\tan^{2}\eta_{c}\sin^{2}\beta_{n}}}\\ K_{\mathrm{AC}}=\frac{\tau_{s}}{\sin\phi_{n}\cos\lambda_{s}}\frac{\sin\left(\beta_{n}-\gamma_{n}\right)}{\sqrt{\cos^{2}\left(\phi_{n}+\beta_{n}-\gamma_{n}\right)+\tan^{2}\eta_{c}\sin^{2}\beta_{n}}} \end{array}\right.$$

in which *β*_n_ stands for the normal friction angle; *ϕ*_n_ represents the normal shear angle; *τ*_s_ is the shear stress; *λ*_s_ is the tool inclination angle (equal to the helix angle for end mills); *η*_c_ is the chip flow angle; *γ*_n_ is the normal working rake angle. Based on Stabler’s chip flow rule, *η*_c_ = *λ*_s_.

Transforming the elemental forces from the tool coordinate frame to the fixed *O*-*XYZ* coordinate frame yields:

(22) $$\left[\begin{array}{c} dF_{xT}\\ dF_{yR}\\ dF_{zA} \end{array}\right]=R(t)\cdot M(K_{r})\left[\begin{array}{c} dF_{T}\\ dF_{R}\\ dF_{A} \end{array}\right]$$

where *M*(*K*_r_) is the coordinate transformation matrix:

(23) $$M(K_{r})=\left[\begin{array}{ccc} 1 & 0 & 0\\ 0 & \cos K_{r} & \sin K_{r}\\ 0 & -\sin K_{r} & \cos K_{r} \end{array}\right]$$

Finally, integrating the transformed elemental forces for all teeth along the axial direction yields the equivalent milling forces in the *X*, *Y*, and *Z* directions:

(24) $$\left\{\begin{array}{c} F_{x}=\int_{0}^{a_{p}}dF_{xT}\\ F_{y}=\int_{0}^{a_{p}}dF_{yR}\\ F_{z}=\int_{0}^{a_{p}}dF_{zA} \end{array}\right.$$

## 4. Simulation Model

To further investigate how axial ultrasonic vibration affects cutting forces, material deformation, and chip formation during titanium alloy milling, and to provide corroborative validation for the proposed analytical model, a 3D FE-based modeling framework was established using DEFORM-3D v11.0 software. This model incorporates tool geometry, the high-strain-rate plastic behavior of the workpiece material, tool-workpiece friction, and axial ultrasonic vibration loads to simulate the cutting-force evolution, plastic deformation characteristics, and the chip formation process under both CM and UVAM conditions.

In the model construction process, detailed geometric modeling of the cutting tool is carried out. We used an 8 mm-diameter four-flute end mill, and its geometric shape is accurately drawn according to the actual tool parameters, including key parameters such as helix angle, rake angle, and relief angle, to ensure that the model accurately reflects the cutting characteristics of the tool. The tool’s geometric parameters are listed in Table 1.

The workpiece is made of Ti-6Al-4V titanium alloy. Considering that a complete workpiece would significantly increase computational scale, and that the initial tool-entry stage is prone to severe local mesh distortion, the workpiece geometry is appropriately simplified in this study. Only the local material region related to the instantaneous cutting process is retained, and the workpiece is set as a semicircular sector structure. This approach reduces the number of elements in non-cutting regions while retaining the real tooth entry and exit characteristics, thereby improving the efficiency of finite element analysis.

The absolute mesh method is adopted for mesh division. Fine mesh generation is performed in key regions, such as the tool cutting edge and the workpiece cutting layer, with a minimum element size set to 0.003 mm to accurately capture the dynamic evolution laws of stress and strain during the cutting process. Coarser meshes are used in non-critical regions, with a maximum element size set to 0.03 mm, to reduce computational cost and improve solution efficiency. Meanwhile, an adaptive remeshing method is adopted during the cutting simulation process to realize chip separation, which can effectively deal with mesh distortion caused by large deformation and ensure the continuity and accuracy of the simulation. The simulation adopts the default convergence settings of DEFORM-3D, and the cutting force curve during the stable cutting stage shows periodic and stable changes without obvious abnormal oscillations. The redivision process is normal. The DEFORM-3D simulation in this study sets the total stroke to 2 mm and the total number of steps to 4000. Figure 8 illustrates the established FE simulation model.

In the simulation of UVAM, the constitutive model of the workpiece material is used to model the plastic flow and deformation of the material in the cutting zone under complex loads. Since Ti-6Al-4V undergoes high strain, high strain rate, and local high temperature during milling, the flow stress of the material is affected by strain hardening, strain-rate strengthening, and thermal softening simultaneously. To improve the descriptive ability of the simulation model for the cutting process, the Johnson-Cook constitutive model is used to represent the mechanical performance of Ti-6Al-4V. This model can well reflect the plastic deformation characteristics during metal cutting and provides a reliable material model basis for cutting force prediction, stress–strain analysis, and simulating chip formation. Its mathematical expression is:

(25) $$\sigma =\left(A+B\varepsilon^{n}\right)\left(1+C\ln\frac{\dot{\varepsilon }}{{\dot{\varepsilon }}_{0}}\right)\left[1-{\left(\frac{T-T_{0}}{T_{melt}-T_{0}}\right)}^{m}\right]$$

The Johnson-Cook flow stress model incorporates strain hardening (*A*, *B*, *n*), strain rate sensitivity (*C*, *ɛ̇*_0_), and thermal softening (*m*), with *T*_0_ = 20 °C and Tmelt representing the melting point.

During machining, thermal energy is primarily generated by the plastic deformation of the chip layer and the friction at the tool–chip interface. A portion of this energy raises the temperature in the cutting zone, while the remainder is dissipated through heat conduction within the workpiece, chip, and tool, as well as through convective heat exchange with the surroundings. In the Johnson–Cook constitutive model, the thermal softening term (1 − (*T* − *T*_0_)/(*T*_melt_ − *T*_0_)*^m^*) is employed to describe the reduction in material flow stress caused by elevated temperature. The material properties are set according to experimental measurements and relevant literature. The accurate setting of these material properties is of great importance for simulation result fidelity and can truly reflect the mechanical and thermal behaviors of titanium alloy during milling. The constitutive model coefficients of the titanium alloy material used in this experiment are shown in Table 2.

In the contact setting, face-to-face contact is adopted between the tool cutting edge and the workpiece surface to ensure that the contact area can transmit the load stably during the cutting process. In this study, the Coulomb friction model was adopted in the simulation with a friction coefficient of 0.6. According to existing finite-element simulation investigations, the reasonable range for the Coulomb friction coefficient falls within 0.5–0.7 for dry cutting of titanium-based alloys using cemented carbide tools [29]. Hence, the adopted value lies within this acceptable range, which can well characterize the frictional interaction between the tool and workpiece during titanium alloy milling and contribute to improving the prediction accuracy of cutting forces and cutting temperatures. In terms of boundary conditions, full constraints are applied to the bottom of the workpiece to simulate the rigid clamping state in the experiment. In the FE model, the tool is set to rotate about its central axis while undergoing a constant feed along the *X* direction and an axial ultrasonic oscillation along the *Z* direction. The *Y*-direction translation is constrained, thus enabling the simulation of the axial UVAM process.

To more accurately reflect the friction and contact state at the tool-workpiece interface, the metal cutting Usui tool wear model was enabled in the finite element simulation of this study:

(26) $$w=\int apVe^{-b/T}dt$$

where *w* is the wear volume; *T* is the absolute temperature; *p* is the pressure; *V* is the relative speed; *a*, *b* are wear coefficients, with *a* = 1 × 10^−6^ and *b* = 855. This parameter is an empirical constant for finite element simulation of hard alloy cutting tools cutting difficult-to-machine materials, referring to the general values for DEFORM simulation of difficult-to-machine materials.

In summary, a 3D FE modeling approach is established in this work, which simultaneously considers the real helical edge geometry of the tool, the thermo-viscoplastic behavior of Ti-6Al-4V, the tool-workpiece interface friction, and the axial ultrasonic motion. Meanwhile, this paper does not conduct a complete grid-independence analysis. The current grid size is mainly determined based on previous simulation experience, the literature support, computational efficiency, and stability in large-deformation calculations. There has been no systematic comparison of the effects of different grid sizes on milling forces, stress–strain, and chip morphology. Therefore, the finite element simulation in this paper serves as an auxiliary research tool, primarily used to reveal the trends in cutting force changes, stress–strain distribution, and chip formation characteristics during ultrasonic vibration-assisted milling. It also provides auxiliary support for analytical models, but its quantitative accuracy has certain limitations. Subsequent work will systematically conduct sensitivity analyses under different grid sizes to further improve the quantitative prediction capability of the finite element model.

## 5. Experimental Setup and Model Parameter Identification

### 5.1. Experimental Setup and Procedure

Milling experiments were conducted on a VMC850B vertical machining center (Bochi Machine Tool Group Co., Ltd., Baoji, China). The main technical parameters of the machining center are as follows: the max. spindle speed is 8000 r/min, and the positioning accuracy is ±0.008 mm. An axial UVAM system, comprising an ultrasonic signal generator and an ultrasonic tool holder (High Beam Energy Nanotechnology Co., Ltd., Jinan, China), was integrated into the machining center. Real-time acquisition of cutting forces was accomplished using a Kistler 9257B three-component piezoelectric force sensor (Kistler Instrumente AG, Winterthur, Switzerland). The charge signals were amplified using a Donghua signal conditioner (Jiangsu Donghua Testing Technology Co., Ltd., Jingjiang, China), digitized by a data acquisition card, and transmitted to a computer. Figure 9 presents the overall experimental setup.

In this study, the milling test was conducted with an axial cutting depth of *a*_p_ = 1.5 mm and a radial cutting width of *a*_e_ = 4 mm. All milling operations were performed using dry cutting without the application of cutting coolant. A standard four-flute solid carbide end mill with an 8 mm diameter was employed for machining Ti-6Al-4V workpieces (dimensions: 80 mm × 80 mm × 15 mm). The physical morphologies of the tool and workpiece are shown in Figure 10, and their mechanical property parameters are listed in Table 3. To verify the dependability of the experimental outcomes, all process parameter configurations were subjected to triple repetition.

### 5.2. Model Parameter Identification

Based on the oblique cutting force coefficient expression given in Equation (21), *K*_TC_, *K*_RC_, and *K*_AC_ are not only related to *β*_n_, *ϕ*_n_, and *τ*_s_ but also affected by *γ*_n_, *λ*_s_, and *η*_c_. Among them, *γ*_n_ and *λ*_s_ can be determined by tool geometric parameters, and *η*_c_ is approximately equal to *λ*_s_ according to Stabler’s chip flow rule. Therefore, the main parameters to be identified through experiments in the model are *β*_n_, *ϕ*_n_ and *τ*_s_. Figure 11 shows the cutting force components and constraint relations of the cutter tooth under equivalent oblique cutting.

Model parameter identification was carried out based on the aforementioned milling experimental platform. Milling tests were conducted on the VMC850 machining center under various feed settings, and three-directional milling force signals in the workpiece coordinate system were collected in real time using a Kistler 9257B three-component piezoelectric dynamometer (Kistler Instrumente AG, Winterthur, Switzerland). Considering that the tool entry and exit stages are greatly affected by transient impacts and tooth engagement/disengagement, to reduce the interference of non-stationary signals on parameter identification results, force signals with large fluctuations during the entry and exit stages were first eliminated, and only the data from the stable cutting interval were selected for subsequent parameter inversion. In terms of force signal processing, low-pass filtering was employed to filter out high-frequency noise while retaining the force signals near the cutting frequency of the tool teeth. Since the measured milling force is the combined result of multiple cutter teeth and multiple axial micro-elements, it is not feasible to directly utilize it to obtain the oblique cutting parameters of a single micro-element. Therefore, an equivalent micro-force identification method was adopted in this work.

According to the cutter tooth’s instantaneous angular position and the coordinate transformation relationship, we transformed the measured force data from the workpiece coordinate system to the tool local system. Then, the local forces were projected onto the rake-face normal plane *P*_N_ to obtain [*dF*_Tn_, *dF*_Rn_, *dF*_An_]. In oblique cutting, based on the force balance relationship in *P*_N_, the angle *β*_n_ can be directly obtained from the ratio of force components:

(27) $$\beta_{n}=\gamma_{n}+\varphi_{n}$$

where *γ*_n_ = arctan(tan*γ*_r_⋅cos*η*_c_), *φ*n = arctan(*dF*_An_/*dF*_Tn_).

According to the maximum shear stress principle, the resultant force on the shear plane forms a 45° angle with the shear direction. Introducing the intermediate variables *ϕ*_m_ and *ψ*_m_, the following geometric relations hold:

(28) $$\sin\psi_{m}=\sqrt{2}\sin\phi_{m}$$

(29) $$\cos(\phi_{n}+\beta_{n}-\gamma_{n})=\frac{\tan\phi_{m}}{\tan\psi_{m}}$$

Meanwhile, the following equation follows from the geometric constraints inherent in oblique cutting:

(30) $$\sin\phi_{m}=\sin\beta_{i}\sin\eta_{c}$$

(31) $$\tan\beta_{n}=\tan\beta_{i}\cos\eta_{c}$$

where *β*_i_ is the spatial friction angle.

By combining Equations (27)–(31), the normal shear angle *ϕ*_n_ can be solved iteratively.

Finally, *dF*_s_ is obtained by projecting the resultant force onto the shear direction:

(32) $$dF_{s}=\frac{dF_{A}\left[\cos\phi_{m}\cos(\phi_{n}+\varphi_{n})\cos\psi_{m}+\sin\phi_{m}\sin\psi_{m}\right]}{\sin\varphi_{n}\cos\phi_{m}}$$

The shear area *A*_s_ is derived from the IUCT and the micro-element length:

(33) $$dA_{s}=\frac{dz}{\cos\lambda_{s}}\cdot \frac{t_{\mathrm{UD}}^{eff}}{\sin\phi_{n}}$$

Then, the instantaneous shear stress is:

(34) $$\tau_{s}=\frac{dF_{s}}{dA_{s}}$$

*K*_TE_, *K*_RE_, and *K*_AE_ can be estimated from the analytical model in reference [30]:

(35) $$\left\{\begin{array}{l} K_{\mathrm{TE}}=r_{e}\tau_{s}\left(\frac{2\beta_{n}}{\cos\beta_{n}}+\pi \sin\beta_{n}\tan\beta_{n}\right)\\ K_{\mathrm{RE}}=2\sqrt{3}r_{e}\tau_{s}\sin\beta_{n}\\ K_{\mathrm{AE}}=K_{\mathrm{TE}}\sin\lambda_{s} \end{array}\right.$$

where *r_e_* is the tool edge radius.

This article divides the experimental data into a parameter calibration set and a model validation set. The calibration set is used exclusively to identify *β*_n_, *φ*_n_, and *τ*_s_, and does not participate in subsequent model validation. The calibration experiment was conducted under the conditions of *n* = 1200 r/min, *A* = 6 μm, and *f* = 20,010 Hz. By varying the feed per tooth, cutting force signals under different instantaneous chip thicknesses were obtained, and data from the stable cutting interval were selected for parameter back-extraction. Figure 12 shows a set of force signals from this calibration set. Three stages can be identified throughout the entire cutting process: entry, steady-state milling, and exit. During the tool entry stage, the three-directional milling forces increase rapidly with obvious fluctuations. After entering the stable milling stage, the milling forces show relatively stable periodic variation. At the final stage, when the tool gradually exits the workpiece, the force signal shows large transient fluctuations again. Therefore, to enhance the stability and reliability of parameter identification, this paper uses the abrupt change points in the force signals during the tool’s cutting-in and cutting-out phases as boundaries, eliminates the signals during these phases, and selects data from the stable segment, approximately 3 s long, for the inverse calculation of model parameters. Model validation utilizes independent operating conditions that do not overlap with the calibration set. The validation processing parameters are shown in Table 4.

According to the above parameter identification process, the discrete distributions of *β*_n_, *ϕ*_n_, and *τ*_s_ with the effective IUCT can be obtained by calculating the experimental force signals under different instantaneous cutting states. The analysis results show that *β*_n_ and *ϕ*_n_ present obvious nonlinear variation characteristics with $t_{\mathrm{UD}}^{\mathrm{eff}}$, so they cannot be simply described by constant parameters over the entire tooth cutting cycle. To introduce the discrete parameters identified by experiments into the instantaneous milling force model, double exponential functions were used in this work to describe the variation laws of the normal friction angle and normal shear angle with the effective IUCT, respectively. Their expressions are:

(36) $$\left\{\begin{array}{l} \beta_{n}(t_{\mathrm{UD}}^{eff})=a_{1}e^{b_{1}t_{\mathrm{UD}}^{eff}}+c_{1}e^{d_{1}t_{\mathrm{UD}}^{eff}}+C_{1}\\ \phi_{n}(t_{\mathrm{UD}}^{eff})=a_{2}e^{b_{2}t_{\mathrm{UD}}^{eff}}+c_{2}e^{d_{2}t_{\mathrm{UD}}^{eff}}+C_{2} \end{array}\right.$$

where *a*_1_, *b*_1_, *c*_1_, *d*_1_, and *C*_1_ are the double-exponential fitting parameters of the normal friction angle; *a*_2_, *b*_2_, c_2_, *d*_2_, and *C*_2_ are the double-exponential fitting parameters of the normal shear angle.

The nonlinear least squares method was used to fit the discrete data obtained from experimental identification, and the optimal fitting parameters are presented in Table 5.

Figure 13 presents the identified results. It is evident that *β*_n_ decreases with increasing instantaneous chip thickness, while *ϕ*_n_ increases with the increase in instantaneous chip thickness, and their goodness-of-fit R^2^ values are 0.9607 and 0.9756, respectively, indicating reliable fitting results. Compared with the conclusions in reference [31], this variation is within a reasonable range. This indicates that at small chip thickness, the effects of the tool edge, as well as the extrusion and friction at the tool-chip interface, are relatively prominent. With the increase in effective chip thickness, material shear removal gradually dominates, and the interface friction effect per unit thickness weakens, leading to an overall decrease in the normal friction angle. Meanwhile, the material deformation state and shear-plane geometric relationship in the cutting zone are adjusted accordingly, making the normal shear angle show obvious thickness dependence.

The shear stress *τ*_s_ varies slightly with the effective IUCT, and its overall distribution is nearly horizontal. Therefore, it is approximately regarded as a constant in this work, and the average value from all effective sampling points is taken as the model input parameter, obtaining *τ*_s_ ≈ 613 MPa. By comparing with the results reported in reference [32], this value is within the reasonable range of the material shear strength of Ti-6Al-4V titanium alloy under high-strain-rate cutting conditions, which verifies the reliability of the experimental results. Finally, the fitted *β*_n_, *ϕ*_n_, and *τ*_s_ were substituted into the oblique cutting force coefficient expression, thus enabling the dynamic calculation of instantaneous cutting force coefficients during UVAM.

## 6. Results and Discussion

### 6.1. Transient Milling Force

To validate the predictive performance of the developed UVAM force model, this article employs verification conditions that do not overlap with the calibration set described in Section 5.2 for calculation and experimental comparison. Three groups of different process parameters were selected for calculation, and MATLAB programming was used to implement the analytical model. The three-directional milling forces obtained from the analytical model were compared with finite element simulation results, as shown in Table 6. The three working conditions change the ultrasonic amplitude and vibration frequency, respectively, while other machining parameters remain unchanged, to test how well the model performs under different ultrasonic vibration conditions. As can be observed in Table 6, the three-axis milling forces predicted by both the analytical model and FE simulation show obvious periodic variation.

This periodic characteristic mainly comes from the process of cutter teeth cutting into and out of the workpiece successively: after the cutter tooth enters the cutting zone, the instantaneous chip thickness increases gradually, and the milling force rises accordingly; when the cutter tooth cuts out, the milling force decreases accordingly. The comparison of multiple process-parameter groups shows that the maximum relative error of the three-directional average milling force between the analytical model and FE simulation is about 9.2%, and the maximum relative error of the three-directional milling force peak is about 10.64%. The prediction results of the established three-directional milling force analytical model maintain good consistency with the FE simulation results in terms of variation trend and peak magnitude, and the errors are within a reasonable range, which fully proves that the theoretical model is reliable and effective.

To more intuitively evaluate the prediction accuracy of the analytical model, a direct comparison was made between the analytical model’s prediction results and the actual experimental data. Multiple sets of stable cutting phase data under different cutting conditions were selected, and the average milling forces in the *F*_X_, *F*_Y_, and *F*_Z_ directions were calculated, respectively. The relative error between the analytical model’s predictions and the experimental values was also calculated. The results are shown in Table 7, with a maximum error of approximately 11.4%, which is within a reasonable error range.

In terms of the model form, when the axial ultrasonic amplitude is zero, the vibration displacement term disappears. At this point, CM can be regarded as the non-vibration special case in the milling force model established in this work. On this basis, to further reveal the influence of axial ultrasonic vibration on milling load, the force characteristics of CM and axial UVAM are further compared. Under the conditions of feed *f*_r_ = 0.06 mm/r and spindle speed *n* = 1200 r/min, after applying axial ultrasonic vibration with a frequency *f* = 20,010 Hz and amplitude *A* = 6 μm, the three-directional average milling forces decreased from 86.23 N, 119.64 N, and 79.18 N in conventional milling to 73.53 N, 101.13 N and 67.71 N, with reduction rates of approximately 14.73%, 15.47% and 14.49%, respectively.

The results show that within the process parameter range of this study, axial ultrasonic vibration can effectively reduce the three-directional average milling forces. There are two main mechanisms: first, axial vibration changes the instantaneous velocity of the tooth micro-element in the normal direction to the cutting edge, causing periodic contact and separation between the tool and workpiece, and shortening the effective load-bearing time; second, axial vibration is mapped to the normal chip thickness fluctuation through the helical edge geometry, reducing the instantaneous material removal volume and interface friction load in some periods. The combined effect of the two makes the average force of UVAM lower than that of CM. However, the above reduction rates are for the average milling force within a single tooth cycle. The application of ultrasonic vibration does not imply that the instantaneous cutting force is always smaller than that in conventional milling.

To evaluate whether the cutting-force reduction achieved in this study is reasonable, the present results were benchmarked against previously reported UVAM investigations. Han et al. [33] investigated high-speed UVAM of titanium alloy and reported cutting forces could be reduced by 32.6% to 35.3% relative to CM. Yang et al. [34] examined longitudinal UVAM of Ti-6Al-4V and found peak reductions of 11.77%, 15.52%, and 17.66% in *F*_X_, *F*_Y_, and *F*_Z_, respectively. Ni et al. [26] performed workpiece UVAM experiments on Ti-6Al-4V and documented reductions of 21.5% to 37.24% in the radial cutting force and 31.02% to 46.30% in the feed-direction cutting force compared with conventional milling. The variation in reported reduction amplitudes among different studies is mainly governed by the vibration mode, vibration parameters, and machining conditions. In the current study, axial UVAM produced reductions of approximately 14.73%, 15.47%, and 14.49% in the three force directions. These values agree well with literature results and lie within the reasonable range reported for UVAM of titanium alloys. This result further substantiates the effectiveness of axial UVAM in reducing machining loads.

In light of the above observations, the effects of machining parameters—namely spindle speed *n*, feed *f*_r_, vibration frequency *f*, and amplitude *A* on the milling force were further investigated. The roles of UVAM machining parameters in affecting milling force were explored by the single-factor control method. Table 4 summarizes the parameter configurations for the single-factor experimental design.

Owing to the interrupted cutting regime in milling, the cyclic contact between the milling cutter and the workpiece engenders substantial alterations in the cutting force. This leads to fluctuating forces encountered during milling, so the average values are usually calculated as the evaluation criterion. Under different machining parameters, the variation trends of the three-directional milling forces are basically consistent, with only differences in numerical values. To streamline the analysis content, this work only conducts an in-depth analysis on the *Y*-direction milling force to study the role of individual process parameters in milling force. The milling force responses to varying UVAM parameters are depicted in Figure 14. The error bars in the figure represent the standard deviation of the results from three repeated experiments under each set of process parameters.

The variation in UVAM force with spindle speed is plotted in Figure 14a. Both simulation and experiment were carried out in the spindle speed range of 600 r/min to 1800 r/min, with all other parameters kept consistent. The results show that with increasing spindle speed, the milling force first increases and then decreases. At lower spindle speeds, a rise in rotational speed brings about an elevated cutting speed, which enhances the strain-rate strengthening effect of the material and increases the *R*(t) to prolong the effective cutting time. Work hardening plays a dominant role, leading to the rise in milling force. With a further increase in spindle speed, the plastic deformation and friction work per unit time increase, leading to a rise in the temperature of the cutting zone. Consequently, the thermal softening effect described by the Johnson-Cook constitutive model becomes more pronounced, partially offsetting strain-rate strengthening and work hardening.

Meanwhile, the tool-workpiece contact rate decreases, and the effective contact time is shortened. These two factors jointly cause the milling force to decline in the high-speed region. This study does not separately quantify the contribution of thermal softening to the reduction in milling force; rather, this trend is attributed to the combined effects of strain-rate strengthening, thermal softening, and variations in contact state. Since the analytical model is mainly constructed based on geometric kinematics and instantaneous chip thickness variation, while the FE simulation and experimental results couple the influences of nonlinear factors such as strain-rate strengthening, thermal softening, and interface friction behavior, there are certain deviations in the amplitude and local trends of milling force among the three, but the overall variation laws are in good consistency.

Figure 14b presents the variation in the UVAM force with feed rate when the feed rate changes in the range of 0.04 mm/r to 0.08 mm/r. It is found that as the feed rate increases, all three results show an obvious upward trend. Per the milling force model, the cutting micro-force is closely related to the effective IUCT. With the increase in feed per tooth, the instantaneous chip thickness increases accordingly, leading to increased material removal cross-sectional area and greater shear deformation volume per tooth, and the material shear load and the tool-chip interface friction load increase correspondingly. Therefore, the three-directional average milling forces increase at higher values of feed per tooth. In addition, the amplitude of the vibration-induced chip thickness component is mainly determined by ultrasonic amplitude, frequency, spindle speed, and helix angle. When the feed per tooth increases, the relative proportion of this vibration component to the total chip thickness gradually decreases, weakening the relative modulation effect of axial ultrasonic vibration on the average milling force. Therefore, the cutting process under large-feed conditions will be macroscopically closer to continuous milling.

The variation in UVAM force with amplitude is shown in Figure 14c. Both simulations and experiments were carried out in the amplitude range of 2 μm to 10 μm, with all other parameters kept consistent. As the ultrasonic amplitude increases, the average milling forces show a downward trend. The core reason is that under the condition of constant vibration frequency, increasing the amplitude enhances the periodic variation in the normal relative velocity of the tool. When the amplitude reaches a range satisfying the separation condition, the *R*(t) decreases, and the effective load-bearing time is shortened. In parallel, the increase in ultrasonic amplitude makes the impact-cutting effect more significant, which promotes the change in material removal form and reduces the resistance during cutting deformation, ultimately reducing the milling force.

Figure 14d shows the theoretical, simulation, and experimental variation laws of UVAM force when the vibration frequency changes in the range of 10,010 Hz to 30,010 Hz. Under elevated ultrasonic vibration frequencies, a gradually decreasing trend is observed for the average milling force. Under the ideal assumption of constant tool-tip amplitude, increasing vibration frequency directly increases the velocity amplitude of axial vibration, making the periodic fluctuation of the normal relative velocity at the tool-workpiece interface significantly enhanced. When the vibration frequency is below a critical threshold, the tool-workpiece interface always maintains a continuous contact state, and the frequency change has not yet changed the cutting contact mode, so the effect on the average milling force is relatively limited. Once the vibration frequency exceeds this critical threshold, the tool-workpiece interface gradually forms significant periodic contact-separation intermittent cutting characteristics; the effective contact duration within a single cutting cycle is shortened, and the interface friction effect is weakened accordingly. The combined effect of the two drives the average milling force to decrease gradually with the increase in frequency.

### 6.2. Stress–Strain and Chip Morphology

With the help of finite element simulation, the internal stress–strain evolution law of the cutting zone, which is difficult to observe directly in experiments, can be intuitively revealed. Significant differences were observed in the material deformation behavior and chip formation characteristics of titanium alloy between CM and axial UVAM. In CM, which is continuous cutting, the milling cutter and workpiece are in constant contact. The material in the cutting zone is subjected to high stress and strain for a long time, which easily leads to accumulated plastic deformation and work hardening, and increases cutting load fluctuation. In contrast, UVAM reduces stress concentration and strain accumulation through high-frequency intermittent contact, making the deformation distribution more dispersed and reducing cutting resistance. In terms of chip morphology, CM tends to produce continuous banded or curled chips, resulting in poor chip evacuation, chip entanglement, and surface scratches. In UVAM, the periodic impact and contact separation of the tool promote chip fracture, making chips short and discrete, and reducing adhesion and friction. It can be seen that UVAM helps to improve chip evacuation, weaken the secondary scratch of chips on the machined surface, and thus improving cutting stability and surface quality.

Figure 15 shows the comparison of equivalent stress contour plots between CM and UVAM. The stress concentration regions for both milling methods are mainly distributed at the contact position between the cutter edge and the workpiece, but the stress concentration range of UVAM is smaller, and its stress peak is slightly lower than that of CM. This suggests that the high-frequency action of ultrasonic vibration can cause periodic micro-separation in the tool-workpiece contact area, thereby effectively reducing the degree of stress concentration during cutting and reducing the instantaneous load at the contact interface. This characteristic helps to alleviate tool fatigue wear, prolong tool life, and reduce residual stress and damage on the workpiece surface, which offers benefits in terms of both surface quality and dimensional stability.

The comparison of equivalent strain contour plots between CM and UVAM is shown in Figure 16. The strain regions of both simulation models gradually expand with the cutting process, but the strain distribution in UVAM is smaller, and the strain peak is lower than that of CM, which is in agreement with the stress distribution law. This is because the high-frequency dynamic impact of ultrasonic vibration can change the plastic deformation behavior of the material, release strain energy more uniformly, inhibit excessive accumulation of local strain, and reduce workpiece plastic deformation. This characteristic helps to reduce machining deformation error, improve machining accuracy, and reduce surface layer strain damage, thereby improving surface integrity and mechanical properties.

The above changes in stress and strain states are ultimately reflected in chip formation and fracture behavior. Table 8 shows the comparison of chip morphology under different machining methods. From the macroscopic morphology characteristics of cutting, the chips produced by conventional milling are thick, continuous, highly curled, and obviously adhered, which easily form long curly chips. This is because the milling cutter and workpiece are always in contact during the cutting process; the chips are continuously plastically extended, and the heat accumulation in the cutting zone aggravates the adhesion between chips and the rake face.

In contrast, the chips from UVAM are fine, short, discontinuous, and have no obvious adhesion. The mechanism is that the high-frequency intermittent cutting effect causes cyclical tool-workpiece disengagement; the chips are broken many times during formation, and the cutting heat dissipates quickly, reducing adhesion and achieving a good chip-breaking effect. The improvement of chip morphology helps to avoid long chips entangling the tool and scratching the machined surface, reduce the secondary friction of chips on the cutting area, and help improve surface quality and inhibit tool wear.

### 6.3. Surface Roughness Study

Surface roughness is a key criterion for judging the surface quality of machined components, which directly affects the wear resistance, fatigue strength, and the fitting accuracy of parts. To evaluate the role of axial ultrasonic vibration in determining the milling surface quality of titanium alloy, the surface roughness of machined surfaces by CM and axial UVAM was compared and analyzed in this section. The surface roughness measurement platform is shown in Figure 17, and the instrument used was a TimeSurf TR300 surface roughness tester (Beijing TIME High Technology Ltd., Beijing, China). During measurement, the machined surface was detected along the feed direction. Three measurement points were selected for each set of process parameters, and the surface roughness for each condition represents the average of three measurements.

Figure 18 plots the surface roughness curves measured under two processing modes. Surface roughness measurements were carried out under the same cutting parameters of feed *f*_r_ = 0.07 mm/r and a spindle speed *n* = 1200 r/min. It was found from the results that the average surface roughness Ra under conventional milling was about 0.314 μm, while after applying axial ultrasonic vibration (*f* = 20,010 Hz, *A* = 6 μm), the surface roughness decreased to about 0.276 μm, with a reduction of approximately 12.1%. Figure 19 compares the variation laws of surface roughness for CM and UVAM at various feed rates. From Figure 19a, it can be noted that at higher spindle speeds, the surface roughness of both machining methods shows a downward trend; Figure 19b reveals a monotonic increase in surface roughness with feed. However, under both parameter conditions, the surface roughness of UVAM is lower than that of CM. It can be seen that the surface profile fluctuation amplitude of UVAM is smaller, high-frequency burrs and scratches are significantly reduced, and surface flatness is significantly improved.

The mechanism of UVAM in improving surface roughness primarily manifests in the following aspects. First, the axial ultrasonic vibration superimposed on the tool causes the bottom edge to undergo high-frequency axial reciprocating motion while rotating and cutting, altering the instantaneous contact state between the bottom edge and the workpiece surface. In conventional milling, the bottom edge continuously deforms the machined surface through plastic lateral flow and rebound, forming deep feed marks and plowing grooves. In contrast, ultrasonic vibration enables the bottom edge to periodically detach from the workpiece surface, reducing sustained compression and thereby minimizing plastic deformation and surface scratches. Secondly, axial vibration induces high-frequency fluctuations in the actual cutting depth of the bottom edge through geometric mapping of the helical cutting edge, which facilitates chip fracture and removal while preventing secondary scratching on the workpiece surface. This also enhances heat dissipation, reducing thermo-induced plastic deformation-related surface waviness. Additionally, the ultrasonic high-frequency impact effectively suppresses low-frequency chatter during milling, alleviating the detrimental effects of tool axial vibration on surface flatness, ultimately achieving a more uniform and refined surface microstructure.

## 7. Conclusions

Taking axial UVAM of Ti-6Al-4V as the research focus, this work conducts theoretical, finite element, and experimental studies focusing on the spatial motion of cutter teeth, tool-workpiece contact state, dynamic instantaneous chip thickness, and three-directional milling force prediction. By constructing a contact-state-driven instantaneous milling force model, the influences of axial ultrasonic vibration on milling force, stress–strain in the cutting zone, and chip formation behavior are analyzed, and the influence of different machining process parameters is discussed. The primary findings are summarized below:

Through kinematic analysis, the tool tip trajectory and tool-workpiece contact rate are derived. Combined with the vibration displacement vector, an instantaneous chip thickness model is constructed, and then an analytical milling force model integrating tool geometric parameters, instantaneous chip thickness, and contact rate is established.

A three-dimensional FE model of the UVAM process is established to simulate and analyze the evolution of chip morphology and milling forces, and the single-factor method is adopted to determine how cutting and vibration parameters affect milling forces.

An experimental platform was constructed. The measured three-component cutting forces exhibit good agreement with predictions from both analytical and finite element simulation; consistent trends are observed, and prediction errors fall within acceptable ranges, which verifies the applicability and trustworthiness of the proposed model for characterizing the mechanical behavior in UVAM.

Axial ultrasonic vibration can significantly reduce the three-component cutting forces, decrease stress and strain within the cutting zone, and promote chip fracture. Within the parameter range investigated in this work, milling forces vary regularly with process parameters and can provide a reference for the selection of actual processing parameters.

The surface roughness Ra can be decreased by roughly 12.1% through UVAM. The improvement mechanism originates from the synergistic effects of intermittent cutting, optimized chip evacuation, and chatter suppression.

## Figures and Tables

**Figure 1:**
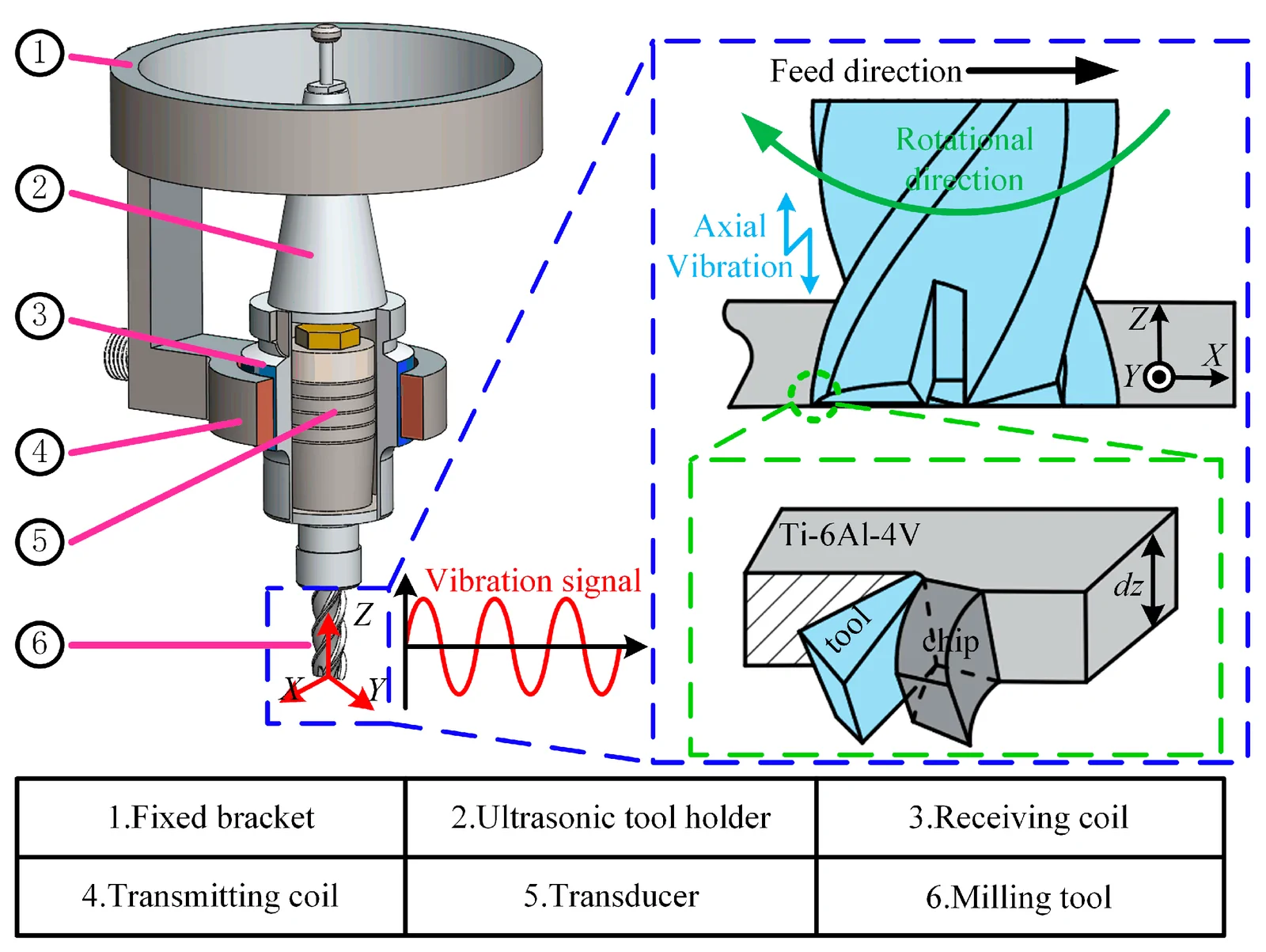
Schematic of the UVAM system and ultrasonic tool holder.

**Figure 2:**
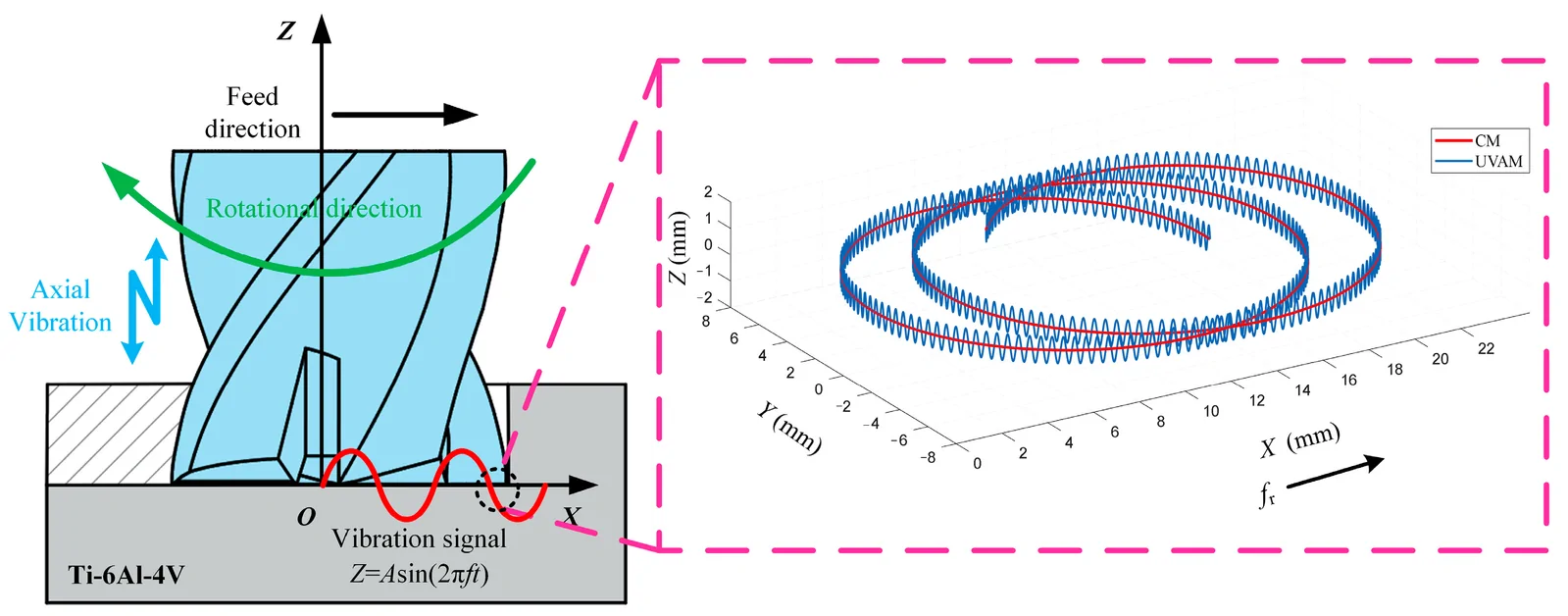
Simulated tool tip trajectories of CM and axial UVAM.

**Figure 3:**
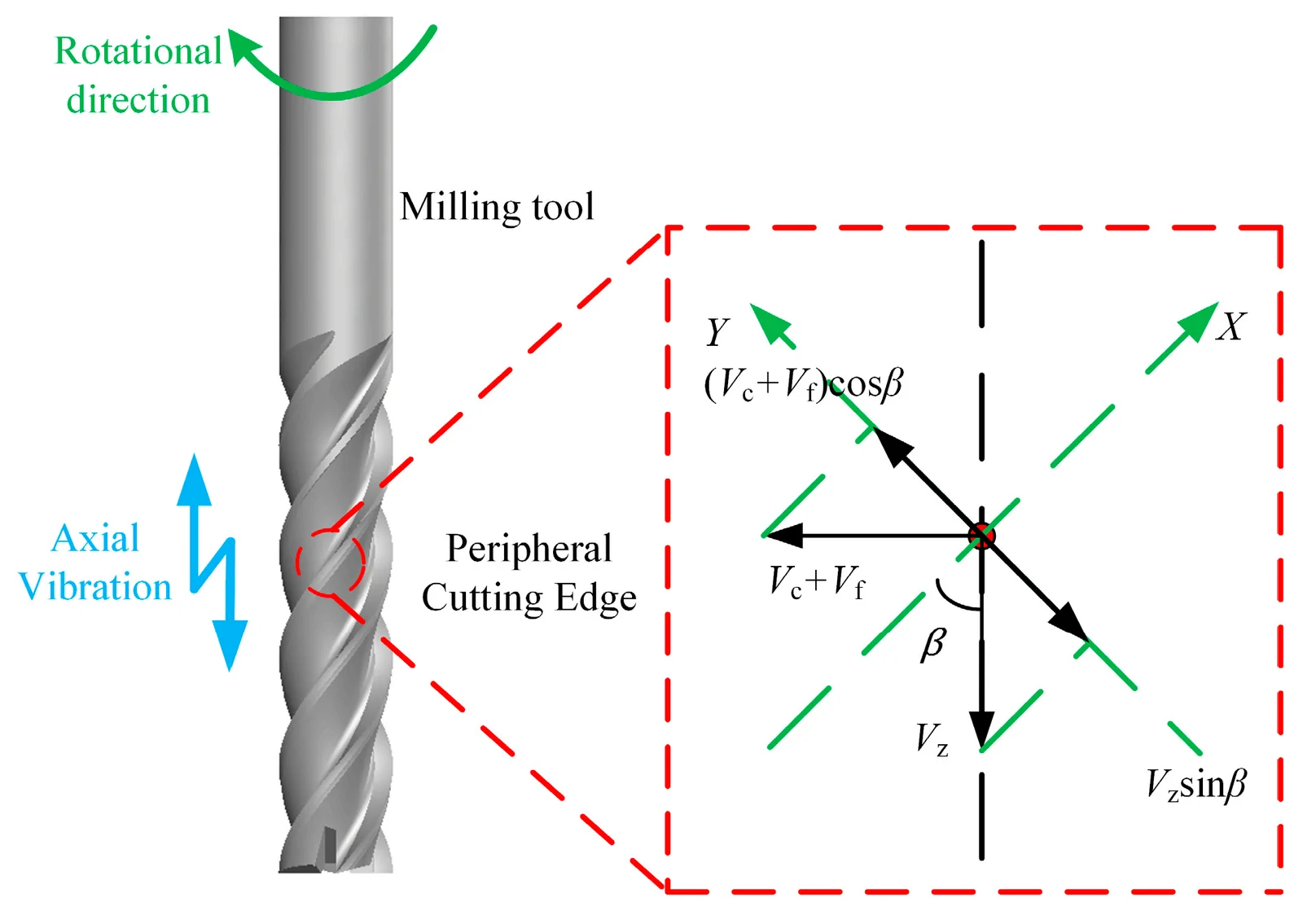
Decomposition diagram of the cutter side edge.

**Figure 4:**
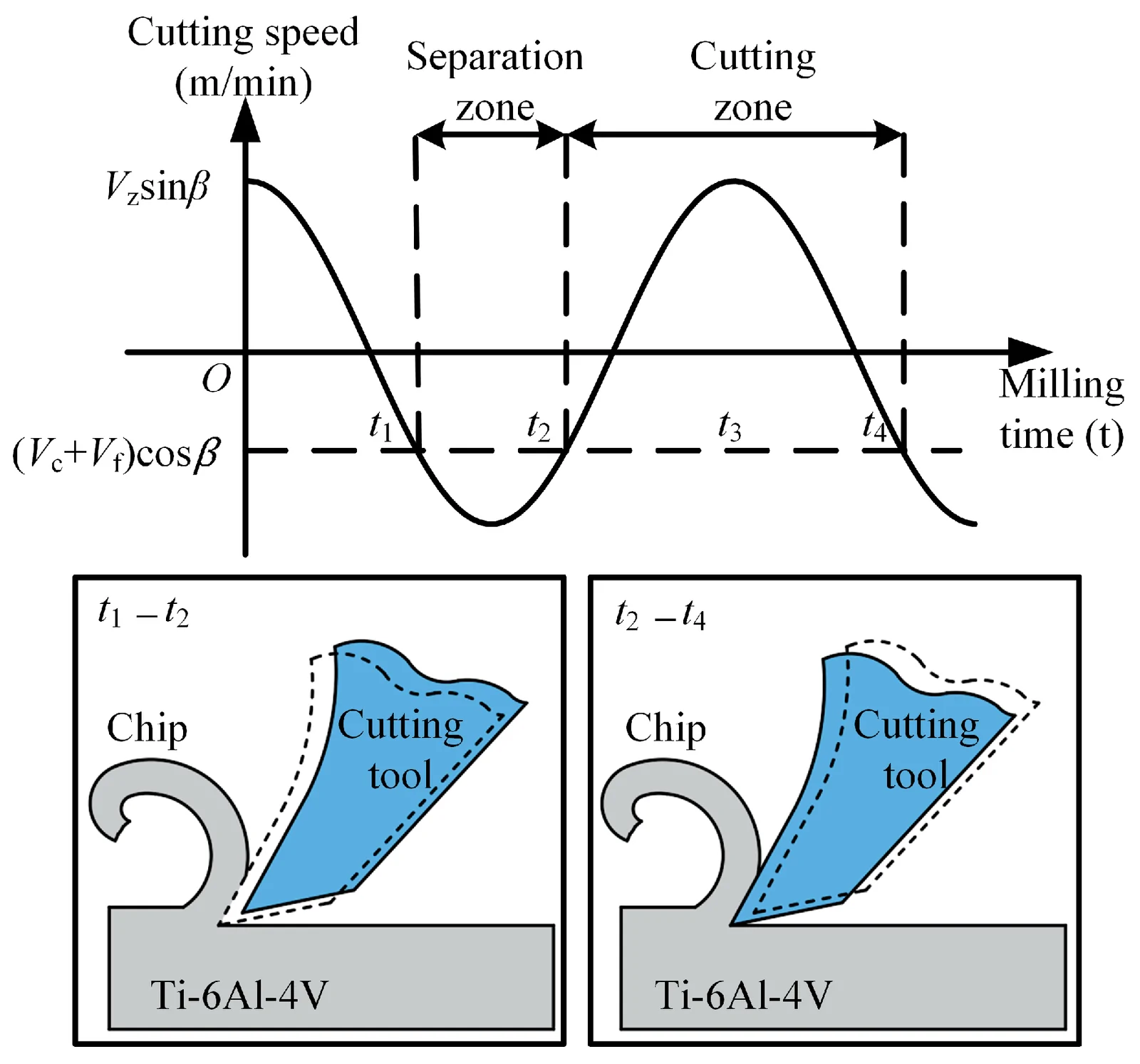
*V*_y_ variation curve and tool-workpiece separation state diagram.

**Figure 5:**
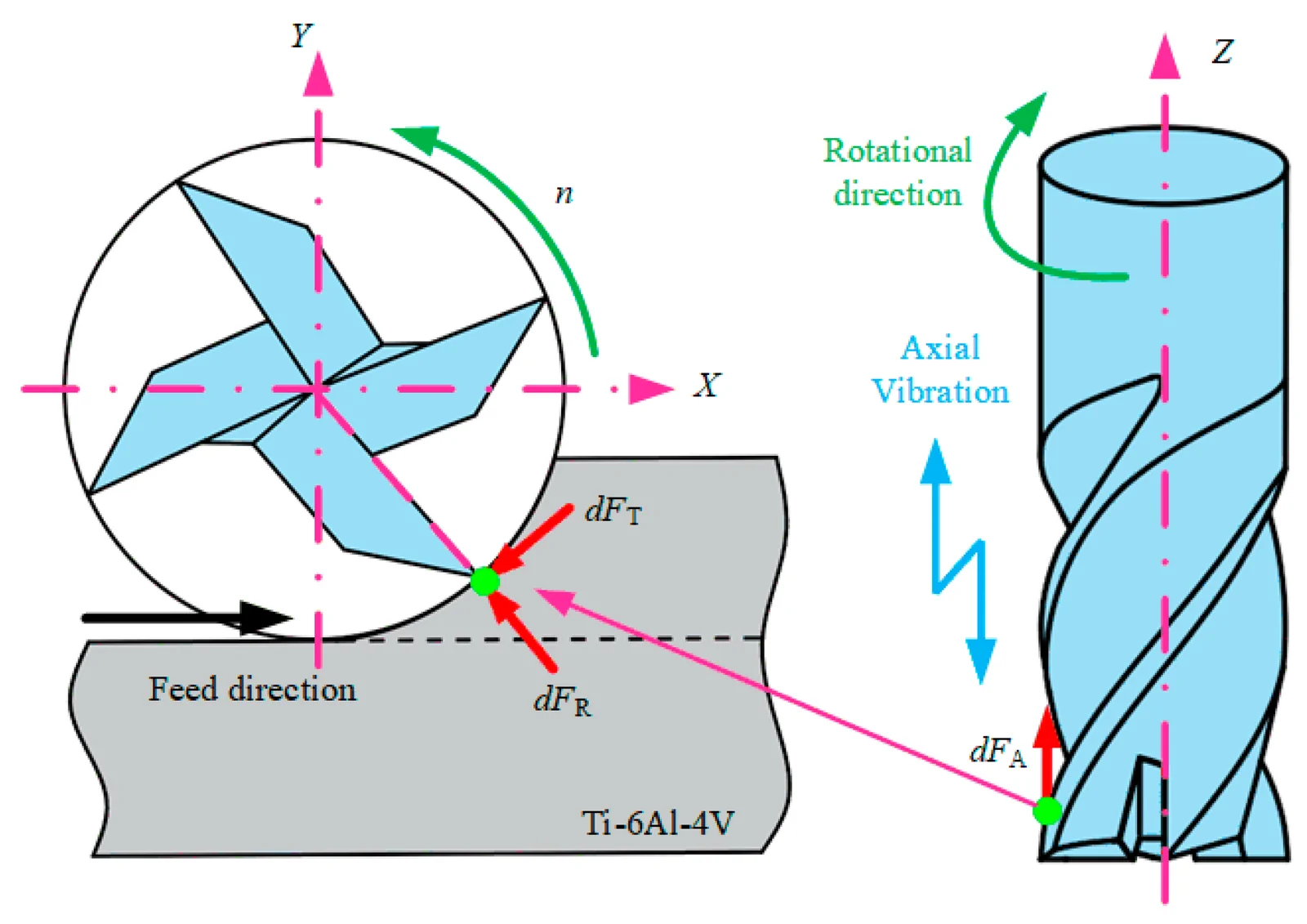
Schematic diagram of cutting forces analysis for the milling tool.

**Figure 6:**
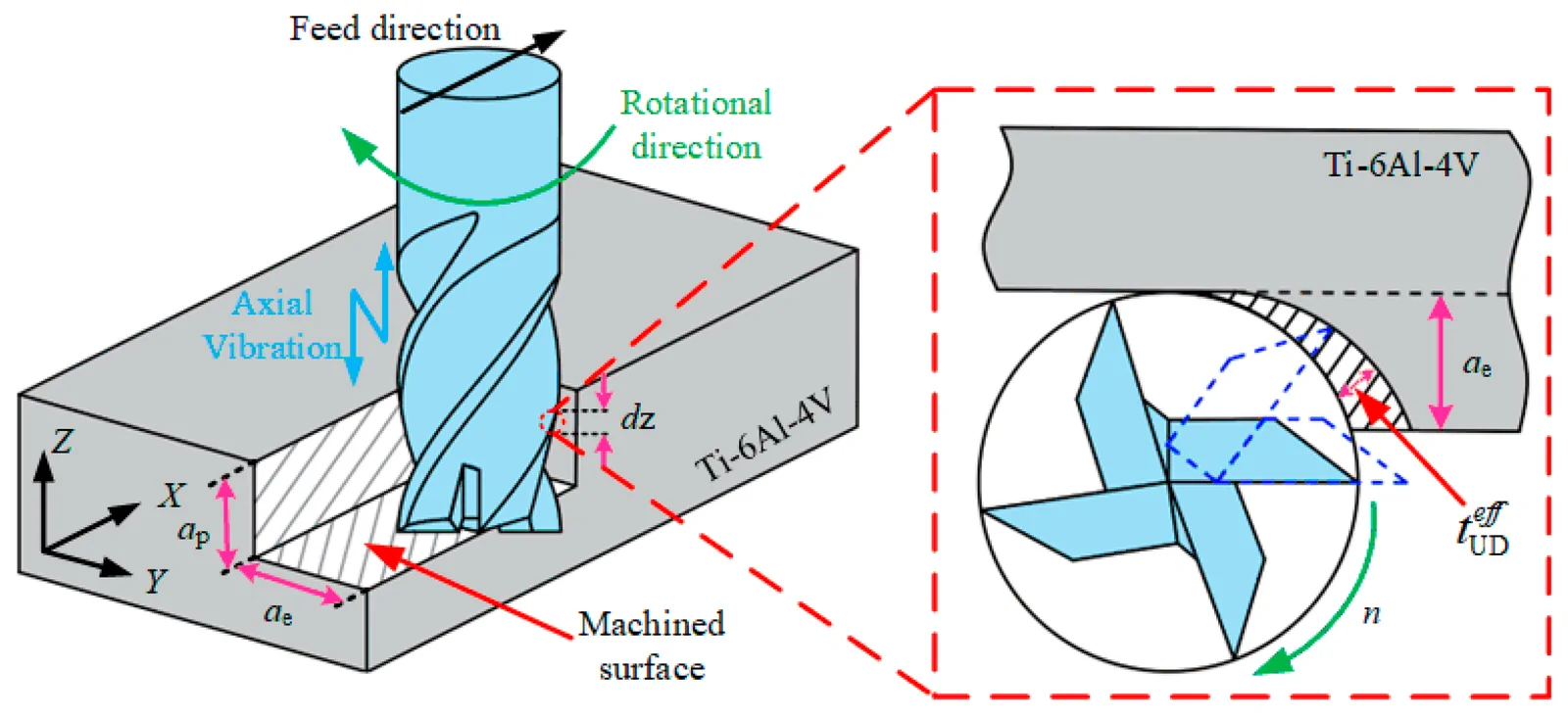
Schematic of the instantaneous uncut chip thickness.

**Figure 7:**
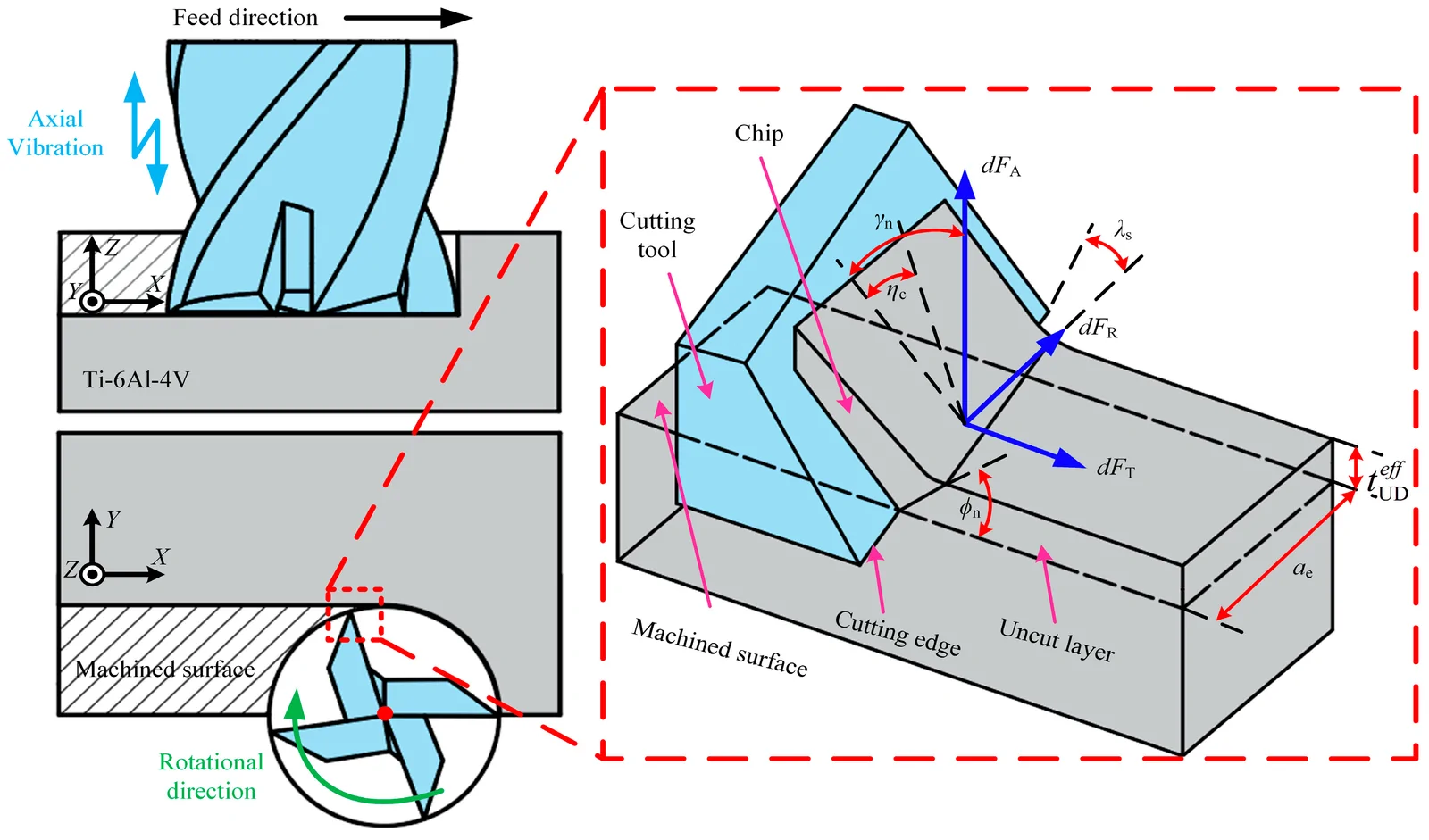
Schematic diagram of oblique cutting machining.

**Figure 8:**
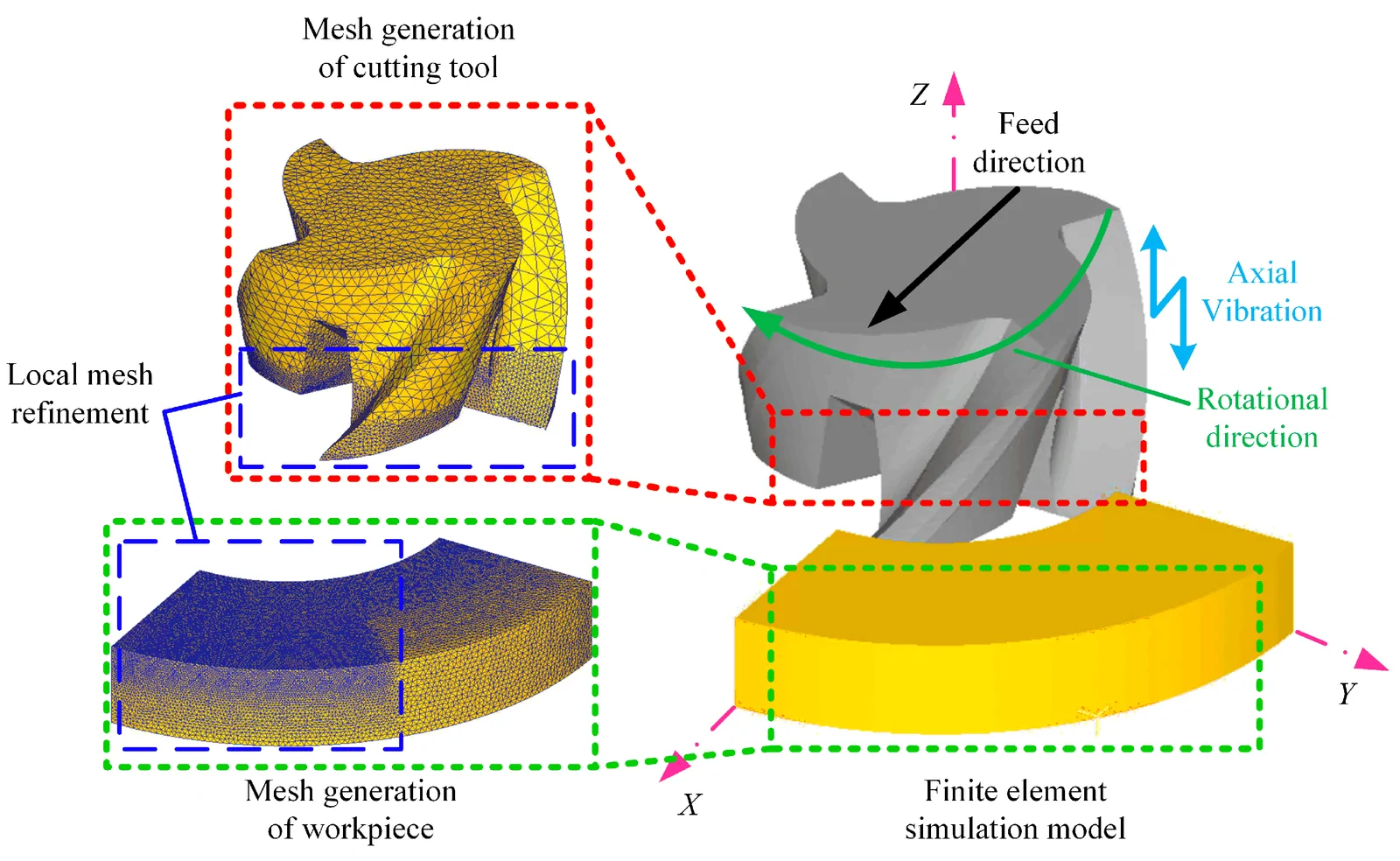
The finite element simulation model.

**Figure 9:**
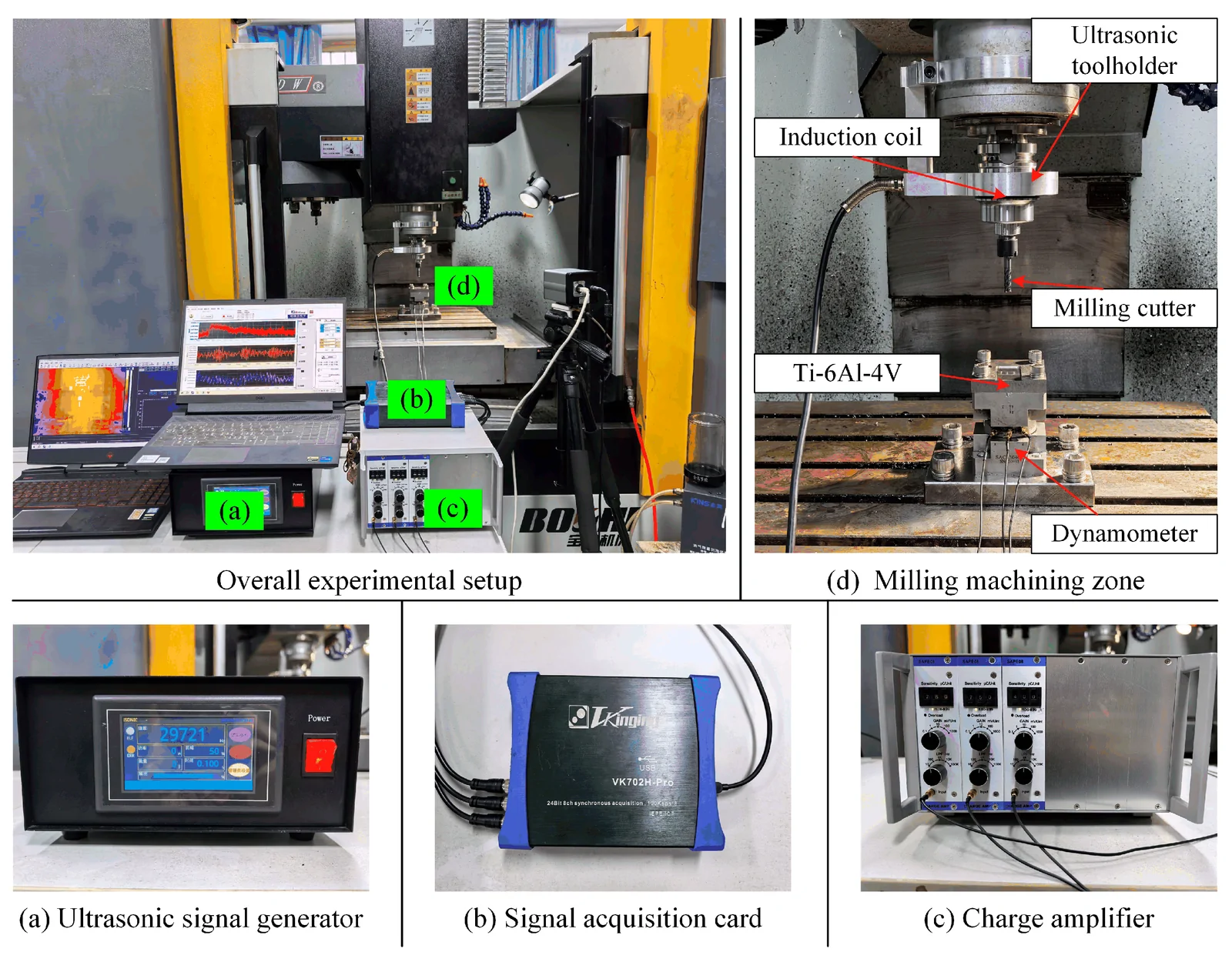
The ultrasonic vibration-assisted milling experimental platform.

**Figure 10:**
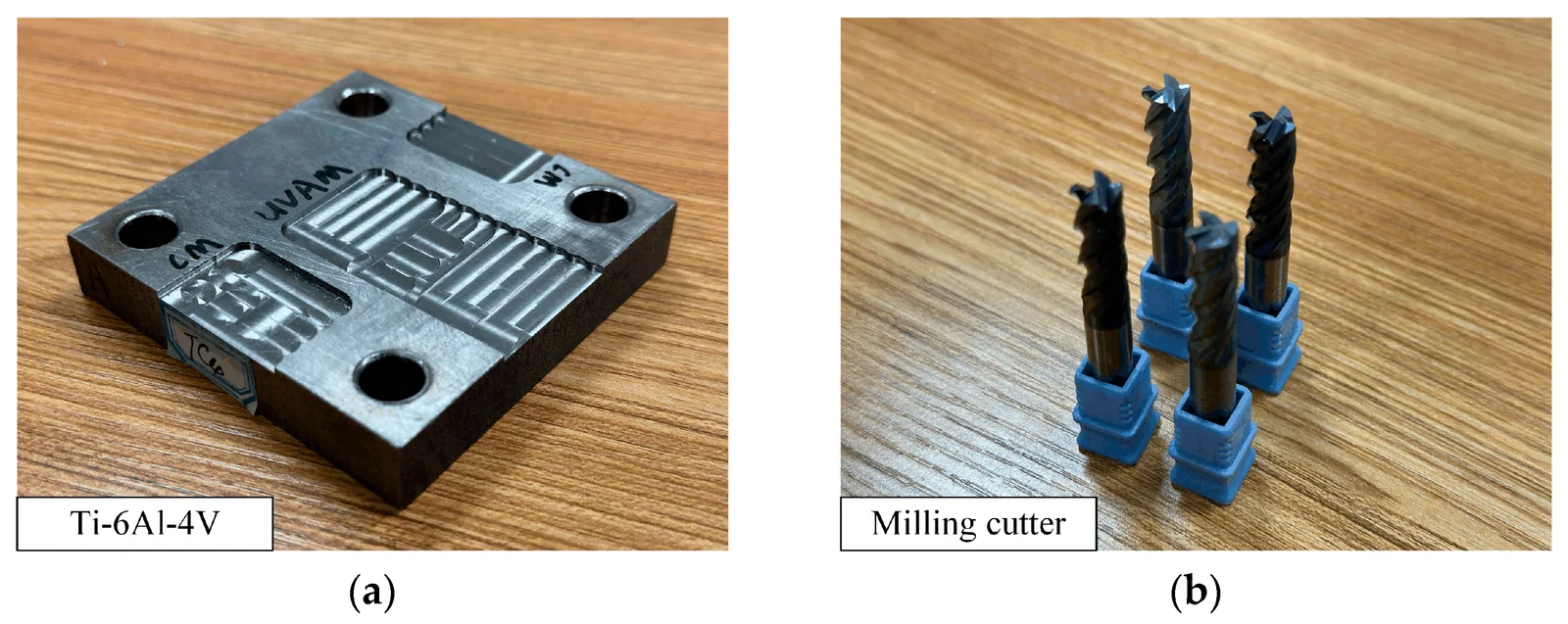
Workpiece material and cutting tool: (**a**) Ti-6Al-4V; (**b**) Milling tool.

**Figure 11:**
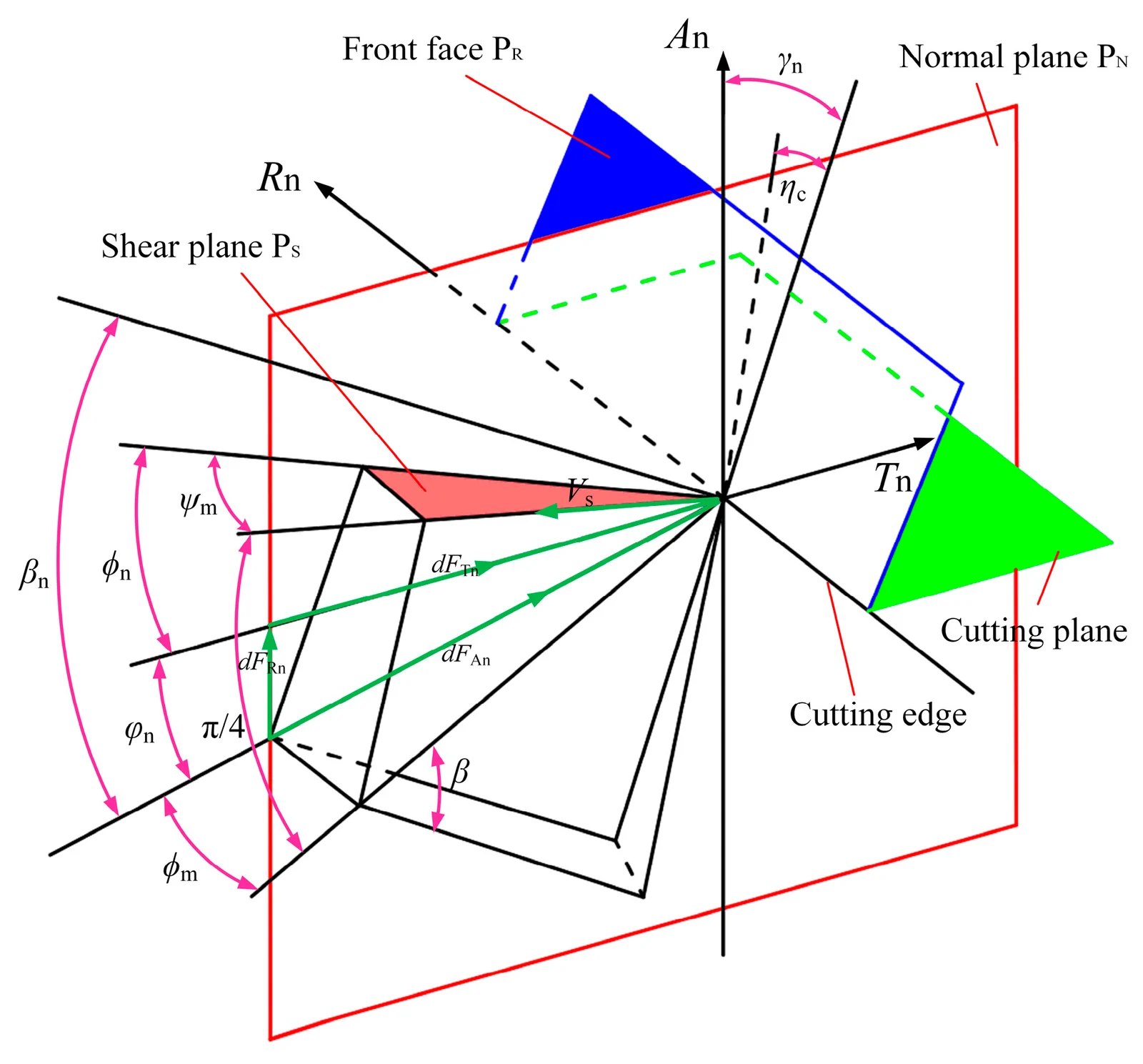
Cutting force components and constraint relations of cutter tooth under equivalent oblique cutting.

**Figure 12:**
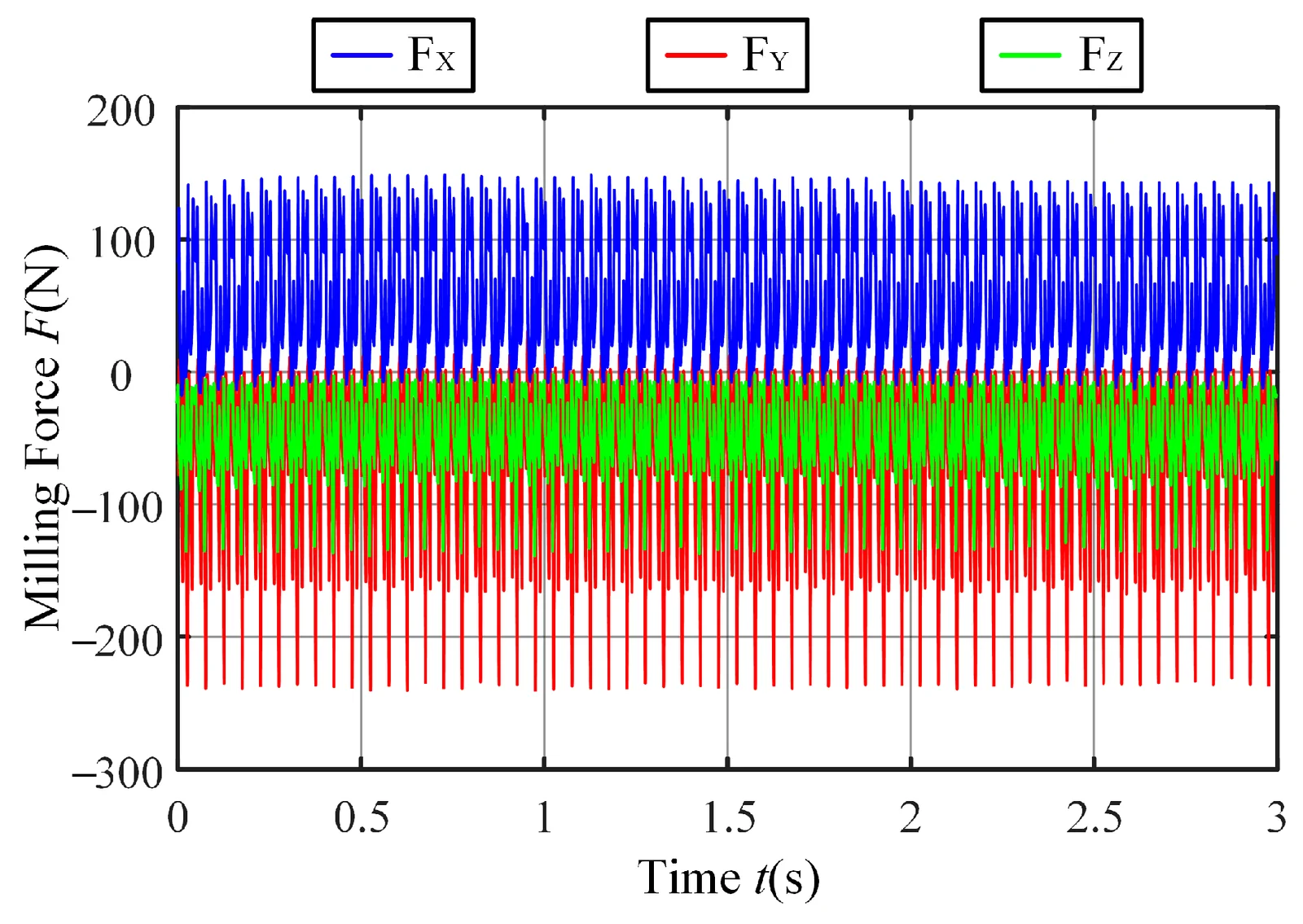
Three-directional milling forces in UVAM experiment under the calibration set.

**Figure 13:**
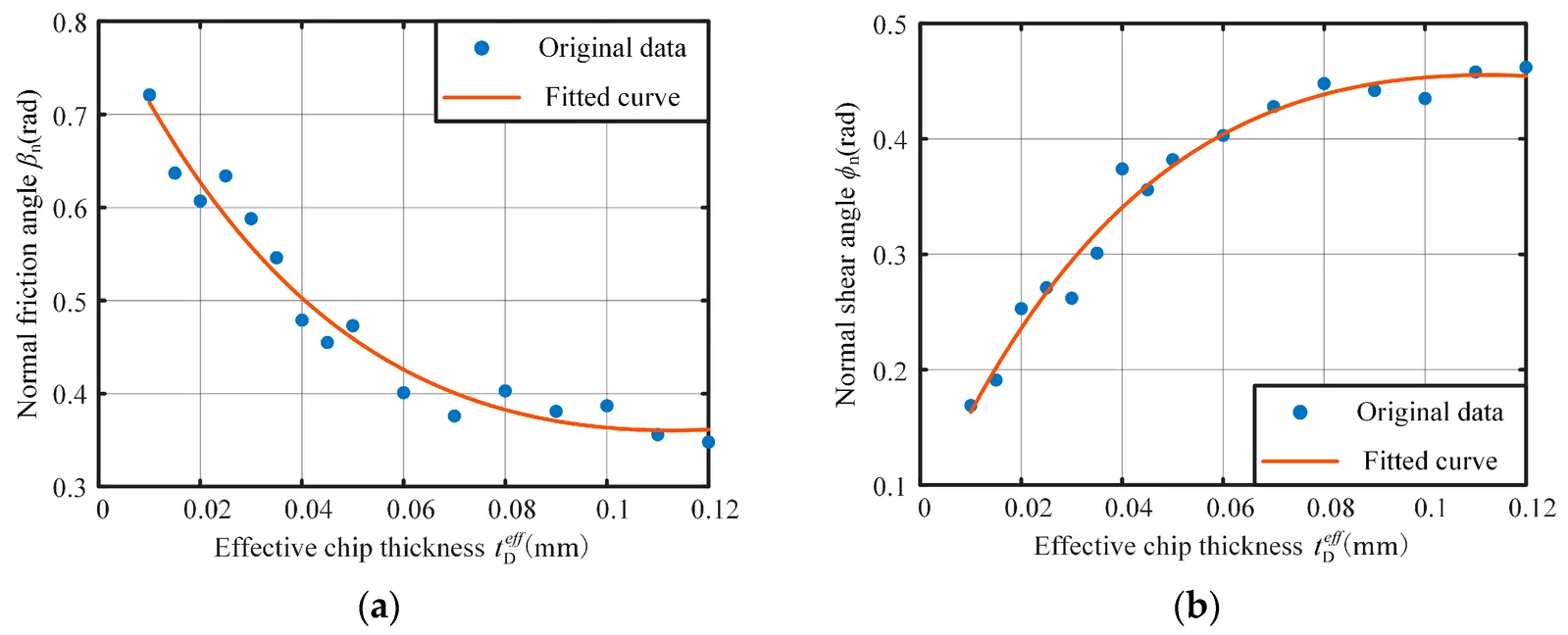
Variation distribution laws of cutter tooth cutting parameters: (**a**) *β*_n_; (**b**) *ϕ*_n_.

**Figure 14:**
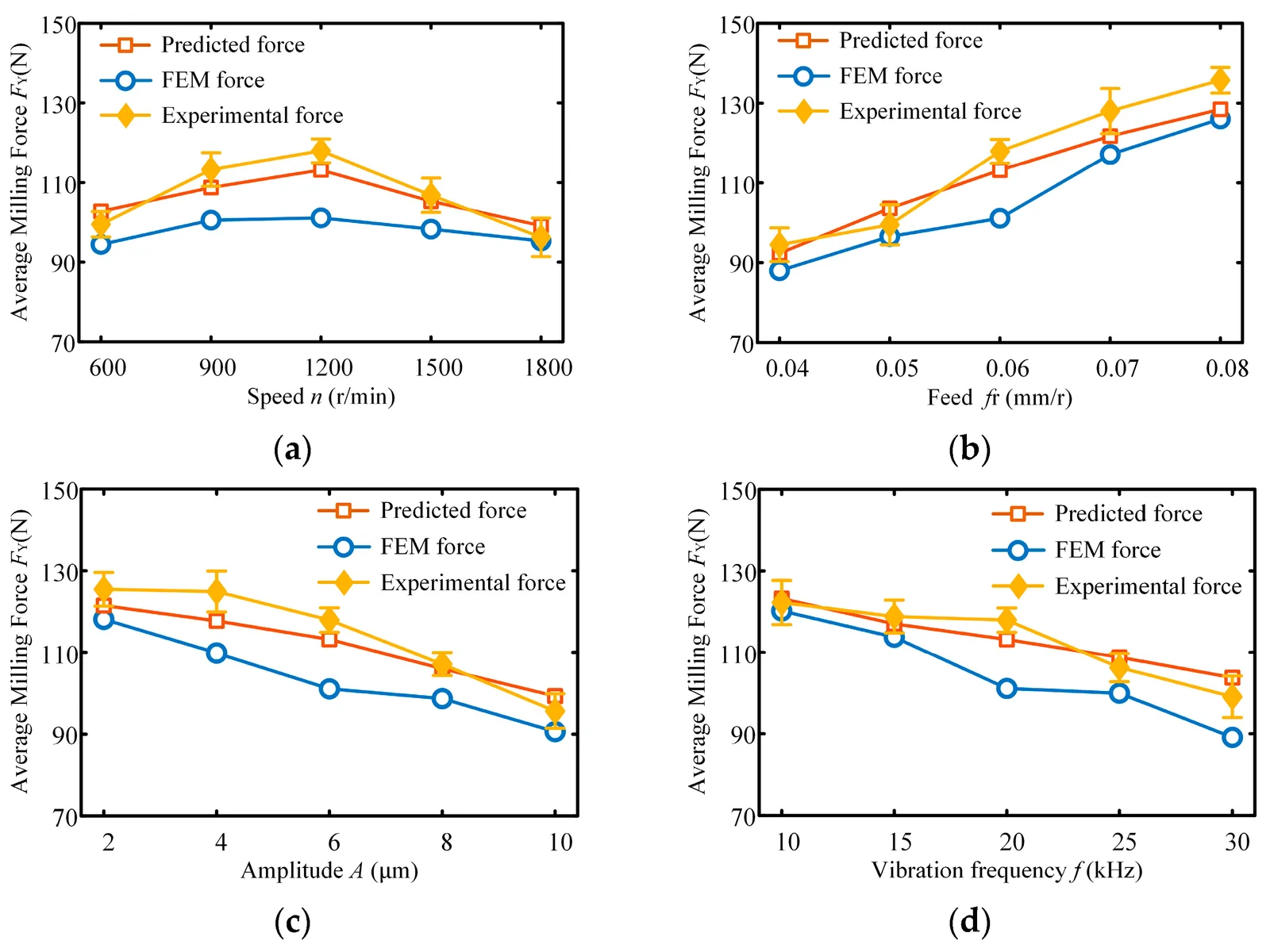
Influence of UVAM process parameters on the Y-direction average cutting force: (**a**) speed; (**b**) feed; (**c**) amplitude; (**d**) vibration frequency.

**Figure 15:**
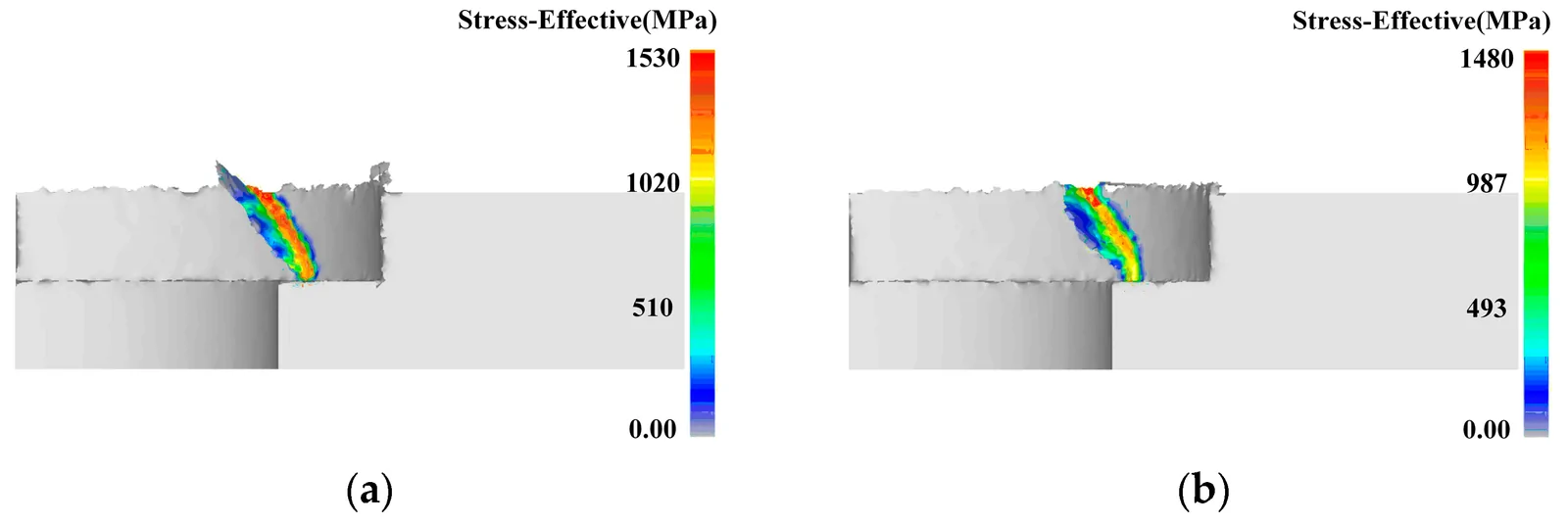
Comparison of effective stress distribution: (**a**) CM; (**b**) UVAM.

**Figure 16:**
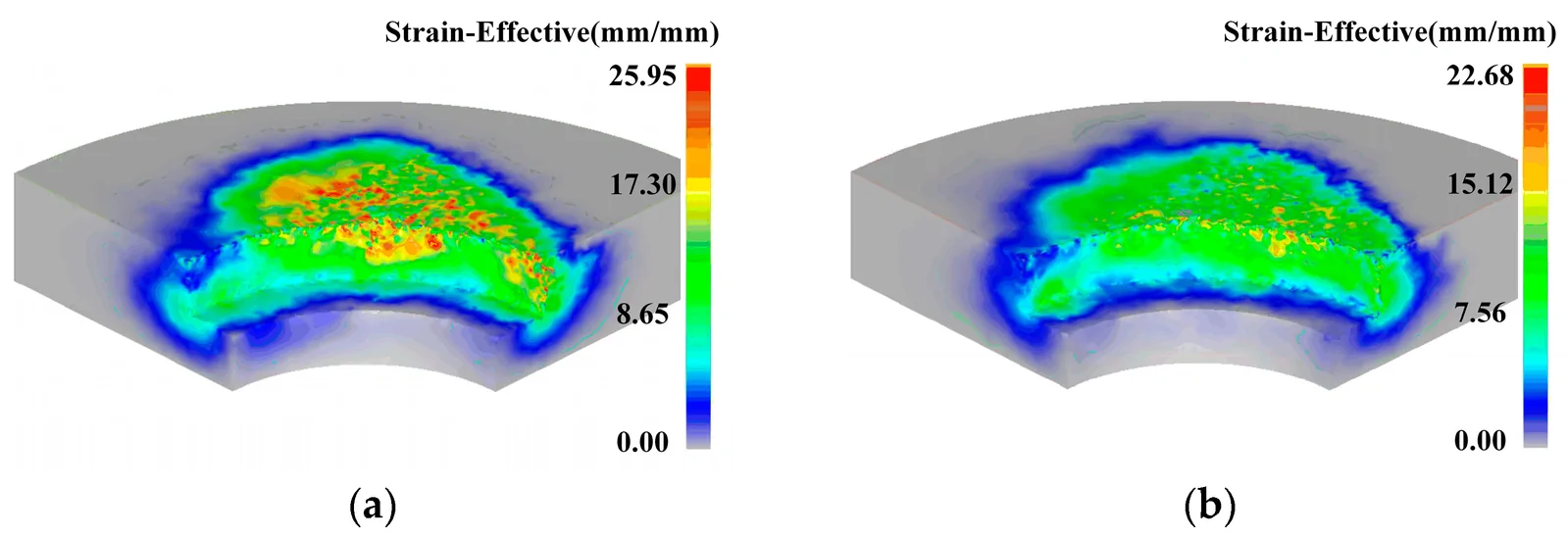
Comparison of effective strain distribution: (**a**) simulated strain contour plot of CM; (**b**) simulated strain contour plot of UVAM.

**Figure 17:**
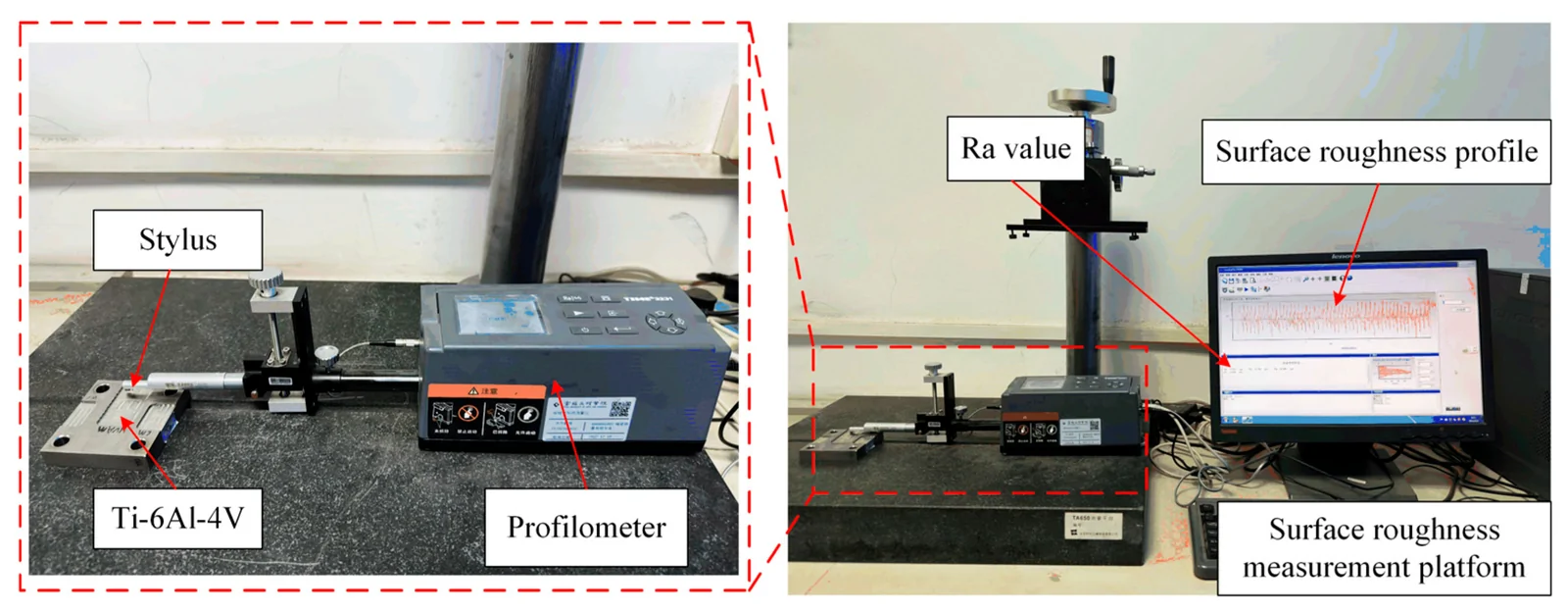
Surface roughness measurement platform.

**Figure 18:**
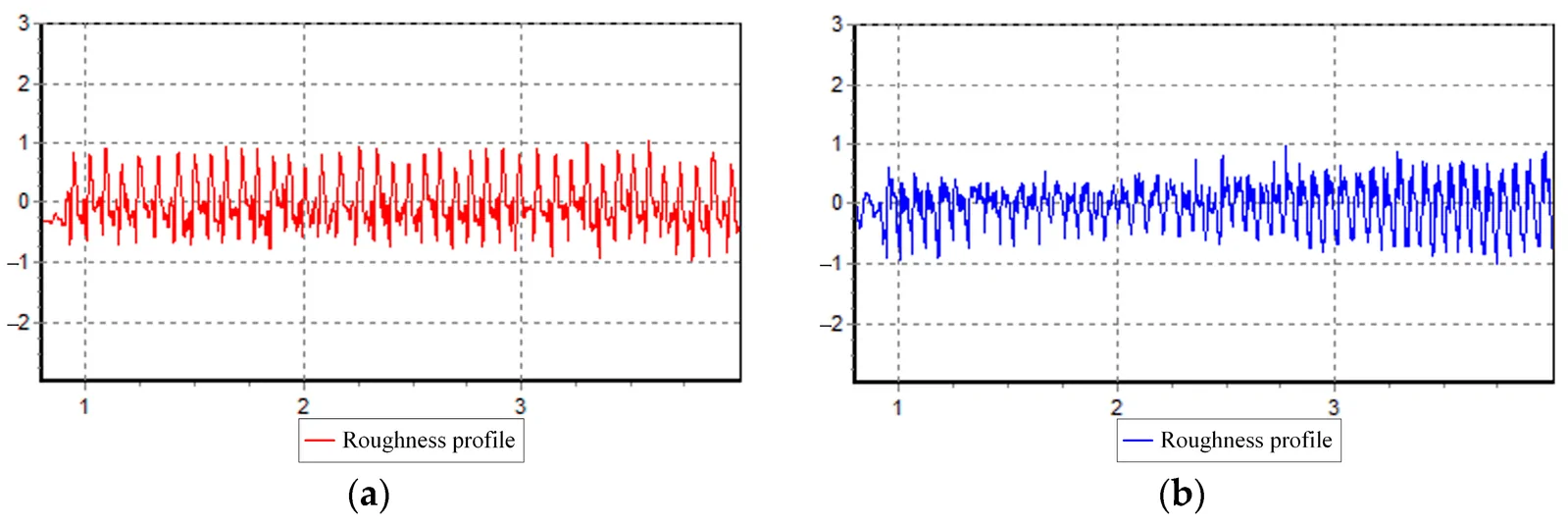
Comparative analysis of surface roughness: (**a**) surface roughness curve of CM; (**b**) surface roughness curve of UVAM.

**Figure 19:**
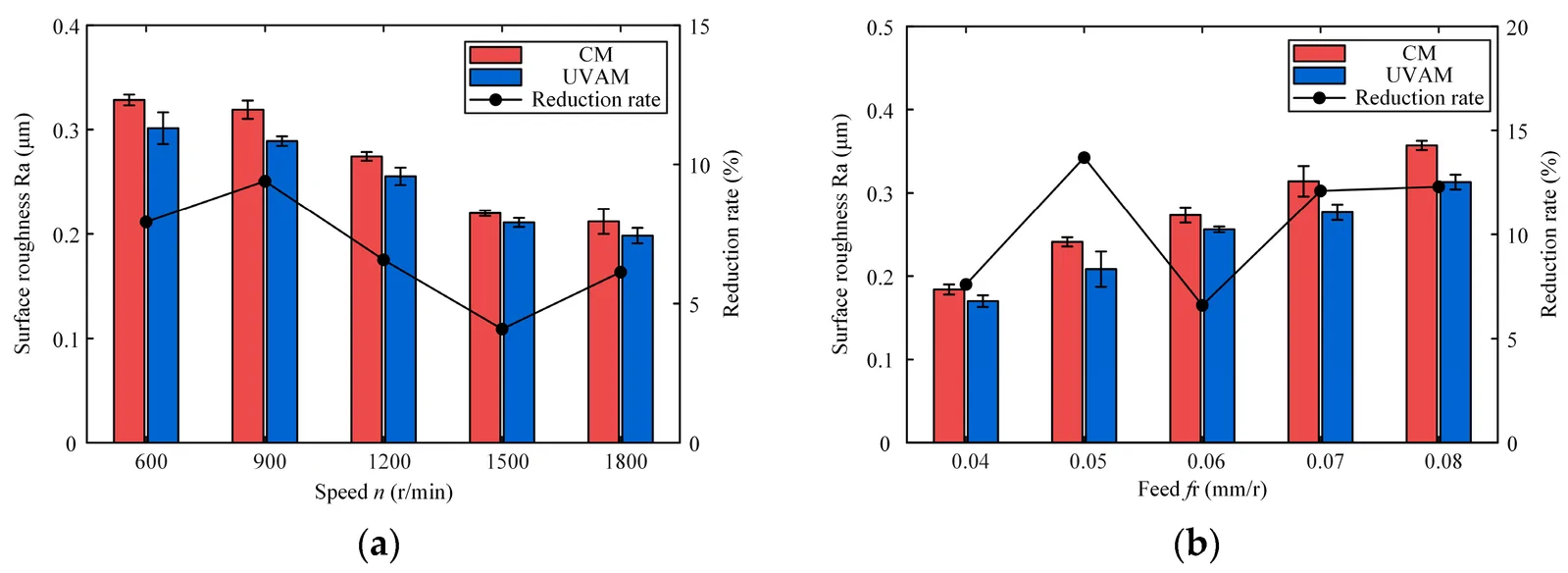
Influence of machining method on surface roughness: (**a**) spindle speed; (**b**) feed.

**Table 1:** Milling tool parameters.

Tool Material	Tooth Count	Diameter/mm	Helix Angle/(°)	Rake Angle/(°)	Relief Angle/(°)
WC	4	8	40	7	12

**Table 2:** Johnson-Cook constitutive model coefficients for Ti-6Al-4V [28].

*A* (MPa)	*B* (MPa)	*C*	*m*	*n*	*T*_melt_ (°C)	*T*_0_ (°C)	${\dot{\varepsilon }}_{0}$
1050	900	0.028	1.0	0.28	1578	20	0.01

**Table 3:** Mechanical parameters of Ti-6Al-4V and WC.

Material	Density *ρ* (g/cm^3^)	Young’s Modulus *E* (GPa)	Poisson’s Ratio *μ*	Hardness (HRC)
Ti-6Al-4V	4.5	113.8	0.342	36~40
WC	14.5	600	0.31	69~81

**Table 4:** Process parameters of ultrasonic vibration-assisted milling.

Parameter	Parameter Levels				
Speed *n* (r/min)	600	900	1200	1500	1800
Feed *f*_r_ (mm/r)	0.04	0.05	0.06	0.07	0.08
Amplitude *A* (μm)	2	4	6	8	10
Vibration frequency *f* (Hz)	10,010	15,010	20,010	25,010	30,010

**Table 5:** Optimal fitting parameters of the double exponential functions.

Fitting Object	*a_i_* (Rad)	*b_i_* (mm^−1^)	*c_i_* (Rad)	*d_i_* (mm^−1^)	*C_i_* (Rad)
*β*_n_	*a*_1_ = 962.451733	*b*_1_ = 0.002076	*c*_1_ = 0.800224	*d*_1_ = −17.016537	*C*_1_ = −962.43403
*ϕ* ^n^	*a*_2_ = 29.131333	*b*_2_ = −10.977196	*c*_2_ = −29.307581	*d*_2_ = −11.248943	*C*_2_ = 0.249892

**Table 6:** Dynamic milling force validation in UVAM.

Parameters	Model Validation (*F*_X_)	Model Validation (*F*_Y_)	Model Validation (*F*_Z_)
*n* = 1200 r/min *f*_r_ = 0.06 mm/r *A* = 6 μm *f* = 20,010 Hz	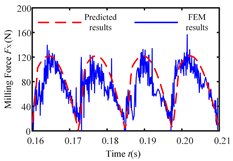	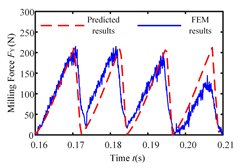	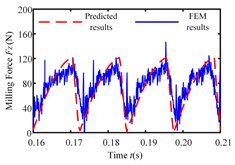
*n* = 1200 r/min *f*_r_ = 0.06 mm/r *A* = 10 μm *f* = 20,010 Hz	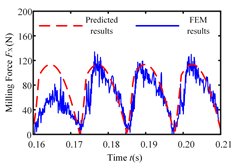	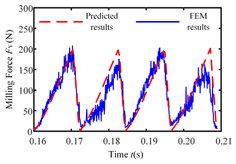	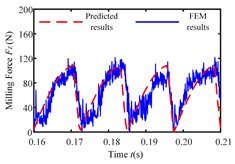
*n* = 1200 r/min *f*_r_ = 0.06 mm/r *A* = 6 μm *f* = 15,010 Hz	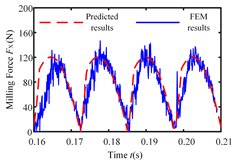	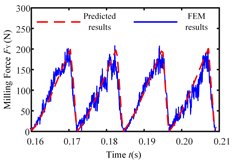	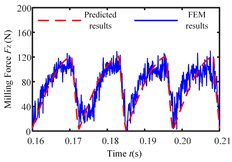

**Table 7:** Comparison and verification of milling forces in UVAM.

Milling Parameters	*F*_i_	Analytical Values	Experimental Values	Relative Error
*n* = 1200 r/min *f*_r_ = 0.06 mm/r *A* = 6 μm *f* = 20,010 Hz	*F*_X_	73.53 N	79.416 N	8.01%
	*F*_Y_	113.19 N	117.852 N	4.12%
	*F*_Z_	67.71 N	72.963 N	7.76%
*n* = 1200 r/min *f*_r_ = 0.06 mm/r *A* = 10 μm *f* = 20,010 Hz	*F*_X_	66.87 N	74.493 N	11.4%
	*F*_Y_	99.34 N	95.687 N	3.68%
	*F*_Z_	64.56 N	60.906 N	5.66%
*n* = 1200 r/min *f*_r_ = 0.06 mm/r *A* = 6 μm *f* = 15,010 Hz	*F*_X_	80.235 N	77.548 N	3.35%
	*F*_Y_	117.02 N	118.831 N	1.55%
	*F*_Z_	74.84 N	81.038 N	8.28%

**Table 8:** Chip morphological comparison.

Classification	CM	UVAM
Simulated chip morphology	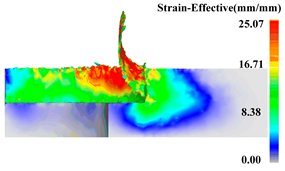	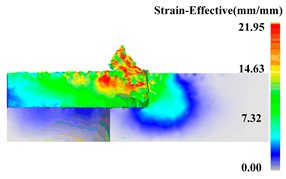
Experimental chip morphology	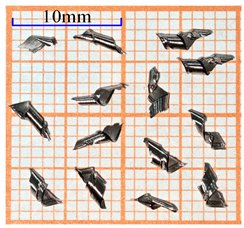	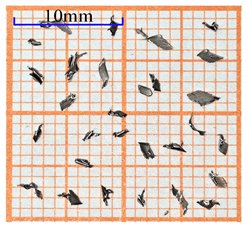

## Data Availability

The original contributions presented in this study are included in the article. Further inquiries can be directed to the corresponding author.
